# MgFe_2_O_4_@Tris magnetic nanoparticles: an effective and powerful catalyst for one-pot synthesis of pyrazolopyranopyrimidine and tetrahydrodipyrazolopyridine derivatives†

**DOI:** 10.1039/d3ra07934a

**Published:** 2024-02-15

**Authors:** Zahra Ramezaninejad, Lotfi Shiri

**Affiliations:** a Department of Chemistry, Faculty of Sciences, Ilam University P.O. Box 69315516 Ilam Iran lshiri47@gmail.com l.shiri@ilam.ac.ir

## Abstract

Magnesium (Mg) as a metal has wide applications, but its use in chemical reactions is rarely reported. Currently, magnesium catalytic processes are being developed to synthesize basic chemical compounds. Therefore, an effective and recyclable nano-catalyst was synthesized using MgFe_2_O_4_@Tris in this study. The structure of MgFe_2_O_4_@Tris was characterized by various techniques including Fourier-transform infrared (FT-IR), scanning electron microscopy (SEM), transmission electron microscopy (TEM), energy dispersive X-ray (EDX), thermogravimetric analysis (TGA), X-ray diffraction (XRD), and vibrating sample magnetometer (VSM) techniques. Finally, the catalytic activity of this nano-catalyst was evaluated for the synthesis of pyrazolopyranopyrimidine and tetrahydrodipyrazolopyridine derivatives. Among the advantages of this catalyst are its high catalytic activity, high yields, use of environmentally friendly solvents, easy magnetic separation, and the possibility of reusing the catalyst.

## Abstract 2

The preparation of pyrazolopyranopyrimidine and tetrahydrodipyrazolopyridine derivatives in the presence of MgFe_2_O_4_@Tris nanoparticles as an innovative catalyst in water and at room temperature has been reported.

## Graphical abstract

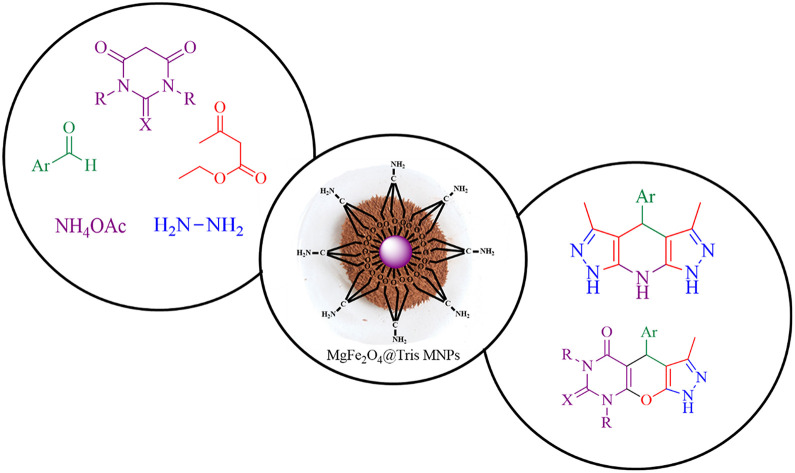

## 1. Introduction

Nanoparticles are an important class of nanometer-scale materials that exhibit unrivaled physicochemical attributes for widespread applications, such as in biomedicine,^1^ biofuels,^2^ sensors,^3^ batteries,^4^ and catalysts.^5^ The increase in the amount of scientific discussion on the topic of magnetic materials shows the increasing interest in this field of science.^6^ Spinel ferrites with the generic formula MFe_2_O_4_ (M = Mg, Mn, Ni, Co, or Zn) are important magnetic substances due to their high stability, excellent electrical and magnetic properties, catalytic properties and biocompatibility.^7^ Magnesium ferrite (MgFe_2_O_4_) is a magnetic nanomaterial that has good magnetic properties and electrical and thermal resistance.^8^ Recently, MgFe_2_O_4_ nanoparticles have attracted increasing amounts of interest because they have various applications in Li-ion batteries,^9^ gas sensors,^10^ catalysts,^11^ and adsorbents.^12^

Multicomponent reactions (MCRs) are reactions between more than three compounds in one step and are important organic chemistry reactions for the synthesis of intricate molecules. They are widely used in all fields of organic synthesis.^13^ In the process of multicomponent reactions, magnetic nanocatalysts are the best option as heterogeneous catalysts due to their easy product separation technique, easy recovery, and environmentally friendly properties.

Heterocyclic compounds^14^ are one of the largest families of organic compounds. They are compounds with a cyclic structure that have at least one carbon atom and at least one heteroatom such as O, N, or S in their structure. Pyrazolopyranopyrimidines^15^ and tetrahydrodipyrazolopyridines^16^ are two important classes of nitrogen-containing heterocyclic compounds with diverse applications. Pyrazolopyranopyrimidines are a group of polycyclic-fused heterocyclic compounds, consisting of rings containing pyrimidine, pyran, and pyrazole. Pyrazolopyranopyrimidine derivatives are of great interest because of their broad pharmacological and biological properties and activities as antimicrobial,^17^ antituberculosis,^18^ anti-inflammatory,^19^ analgesic,^20^ and anticancer agents.^21^ Tetrahydrodipyrazolopyridines are among the most important nitrogen-containing heterocyclic compounds, and they consist of two parts, pyrazole and 1,4-dihydropyridine. Due to anticancer,^22^ antiviral,^23^ and anti-leishmanial^24^ properties, they are of particular importance in organic chemistry. Nanocatalysis has found numerous applications in the synthesis of heterocyclic compounds due to the development of nanotechnology.^25^ A majority of currently available and commercially approved drugs contain heterocycles containing nitrogen and oxygen. Due to the versatile applications of heterocycle synthesis catalyzed by metal nanoparticles, its significance should not be overlooked.^26^

**Scheme 1:**
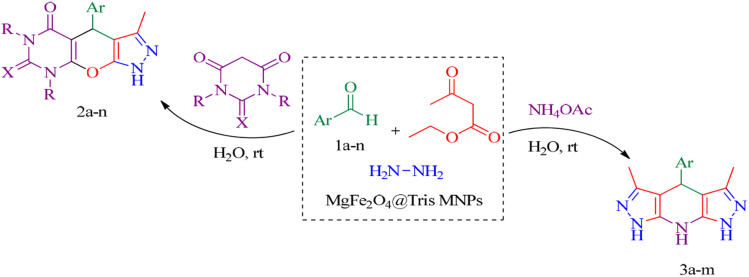
Synthesis of pyrazolopyranopyrimidine and tetrahydrodipyrazolopyridine compounds using MgFe_2_O_4_@Tris MNPs.

In this research, we report tris(hydroxymethyl) aminomethane-functionalized MgFe_2_O_4_ magnetic nanoparticles (MgFe_2_O_4_@Tris) as a new, efficient and recyclable catalyst for the synthesis of pyrazolopyranopyrimidine and tetrahydrodipyrazolopyridine derivatives. These heterocyclic compounds were prepared under mild conditions in a green solvent and at room temperature (Scheme 1).

## 2. Experimental

### 2.1. General

All reactants used in this research were purchased from Merck, Fluka, or Sigma-Aldrich chemical companies. The melting points were defined using a Barnstead Electrothermal 9100 in capillary tubes. The infrared (IR) spectra of samples were recorded in KBr pellets using a VRTEX 70 spectrophotometer (Bruker, Germany). ^13^C and ^1^H NMR spectra (in Hertz) were obtained using a Bruker DRX-250 AVANCE instrument in DMSO-*d*_6_ as the solvent and TMS as the internal standard. Energy-dispersive X-ray spectroscopy (EDX) and scanning electron microscopy (SEM) were carried out and utilized a Czech TESCAN instrument. Thermogravimetric analysis (TGA) was performed using a thermogravimetric analyzer (PerkinElmer-STA6000, USA), and magnetic measurements of the nanocatalyst were obtained using a vibrating sample magnetometer (VSM; MDKB). X-ray diffraction (XRD) was carried out using a Holland Philips PW1730 and TEM of the magnetic nanoparticles (MNPs) was recorded with a Philips-EM 208S TEM.

### 2.2. Synthesis of MgFe_2_O_4_@Tris MNPs

#### 2.2.1. General method for the synthesis of MgFe_2_O_4_ nanoparticles

A mixture of FeCl_3_ (8 mmol = 1.3 g) and MgCl_2_·6H_2_O (4 mmol = 0.81 g) was dissolved in 40 mL of deionized water in a round-bottomed flask (250 mL). Then, it was stirred for 30 minutes at 500 rpm (revolutions per minute) by a mechanical stirrer. Then, 10 mL of sodium hydroxide solution (0.1 M) was added to the reaction mixture and heated for 2 h at 80 °C until brown precipitates were obtained. The product was separated by an external magnet and washed with deionized water, then washed twice with ethanol and dried in an oven at 80 °C for 24 h and calcined at 700 °C for 2 h.^11^

#### 2.2.2. General method for the synthesis of MgFe_2_O_4_@Tris nanoparticles

First, 1 g of MgFe_2_O_4_ produced in the previous step was dispersed in a mixture of 20 mL of H_2_O and 30 mL of EtOH for 20 min. Then, 2.0 g of tris(hydroxymethyl)aminomethane was added and refluxed for 24 h. Finally, the obtained MgFe_2_O_4_@Tris MNPs were separated by an external magnet and washed many times with water and dried at 80 °C.^27^

### 2.3. General method for the synthesis of pyrazolopyranopyrimidine derivatives

Initially, 1 mmol of hydrazine hydrate, 1 mmol of ethyl acetoacetate, and 2 mL of H_2_O were added to the reaction vessel. Then, 1 mmol of aldehyde, 1 mmol of barbituric acid and 10 mg of MgFe_2_O_4_@Tris were added to the reaction mixture, and the resulting mixture was stirred at 300 rpm at room temperature. The progress of the reaction was monitored by thin-layer chromatography (TLC). After completion of the reaction, 10 mL of hot ethanol at a temperature of about 60 °C was added to the reaction mixture to dissolve the solid product. The catalyst was separated using an external magnet. The solvent was evaporated, and the crude product was obtained. The pure product was prepared through recrystallization from hot ethanol. The obtained pure products were characterized using melting points, FT-IR spectra, ^1^H NMR spectra and ^13^C NMR spectra.

### 2.4. General method for the synthesis of tetrahydrodipyrazolopyridine derivatives

In a 5 mL round-bottomed flask, a mixture of ethyl acetoacetate (2 mmol), hydrazine hydrate (2 mmol), aldehyde (1 mmol), ammonium acetate (3 mmol), and MgFe_2_O_4_@Tris (15 mg) in water (2 mL) was stirred at 300 rpm at room temperature for an appropriate amount of time. The reaction progress was followed using thin-layer chromatography. After completion of the reaction, 10 mL of hot ethanol (at about 60 °C) was added to the reaction mixture to dissolve the solid product. Then, the catalyst was separated by a magnet. The solvent was evaporated, and the crude product was obtained. The pure product was prepared through recrystallization from hot ethanol. The obtained pure products were characterized using melting points, FT-IR spectra, ^1^H NMR spectra and ^13^C NMR spectra.

## 3. Results and discussion

### 3.1. Synthesis and characterization of MgFe_2_O_4_@Tris

**Scheme 2:**
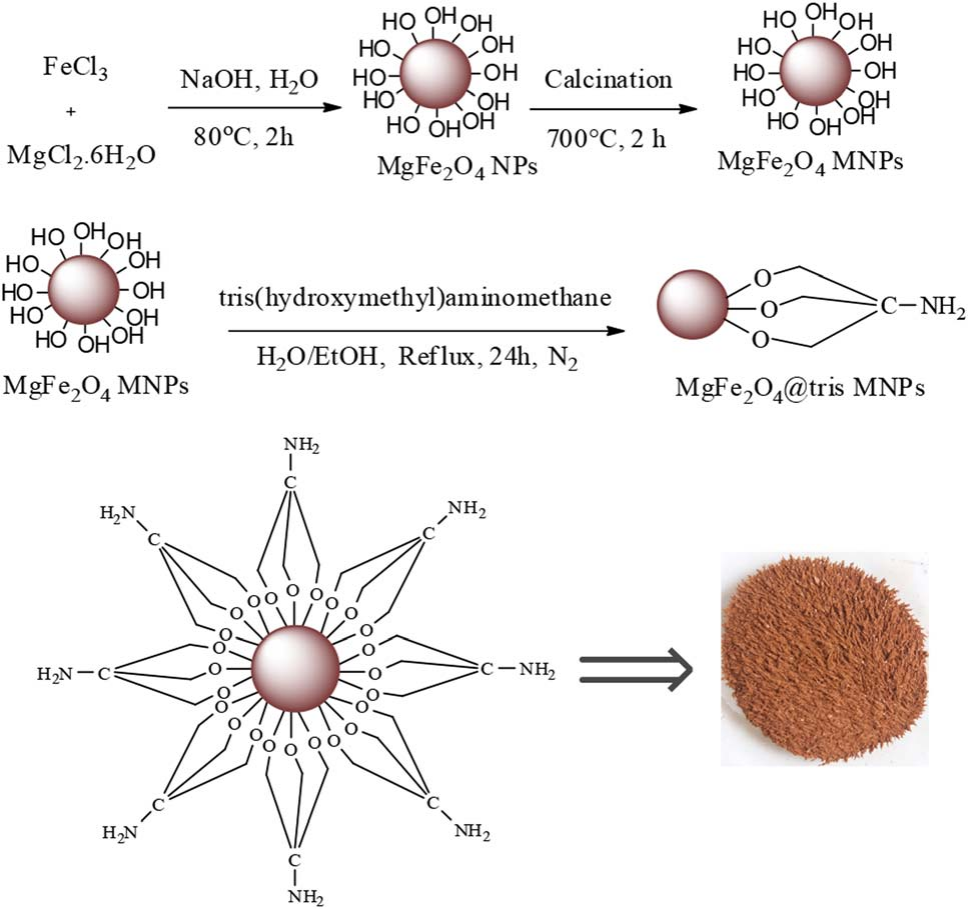
Stepwise synthesis of MgFe_2_O_4_@Tris MNPs.

MgFe_2_O_4_@Tris nanoparticles were successfully prepared by the method outlined in Scheme 2. First, magnesium ferrite MNPs were synthesized using the co-precipitation technique.^11^ Next, MgFe_2_O_4_@Tris was prepared by reacting MgFe_2_O_4_ with tris(hydroxymethyl)aminomethane in refluxed EtOH/H_2_O under nitrogen for 24 h.^27^ Next, the prepared catalyst was fully identified by various characterization methods such as FT-IR, VSM, EDX, EDX mapping, TEM, SEM, XRD, and TGA analysis.

#### 3.1.1. FT-IR analysis

**Fig. 1:**
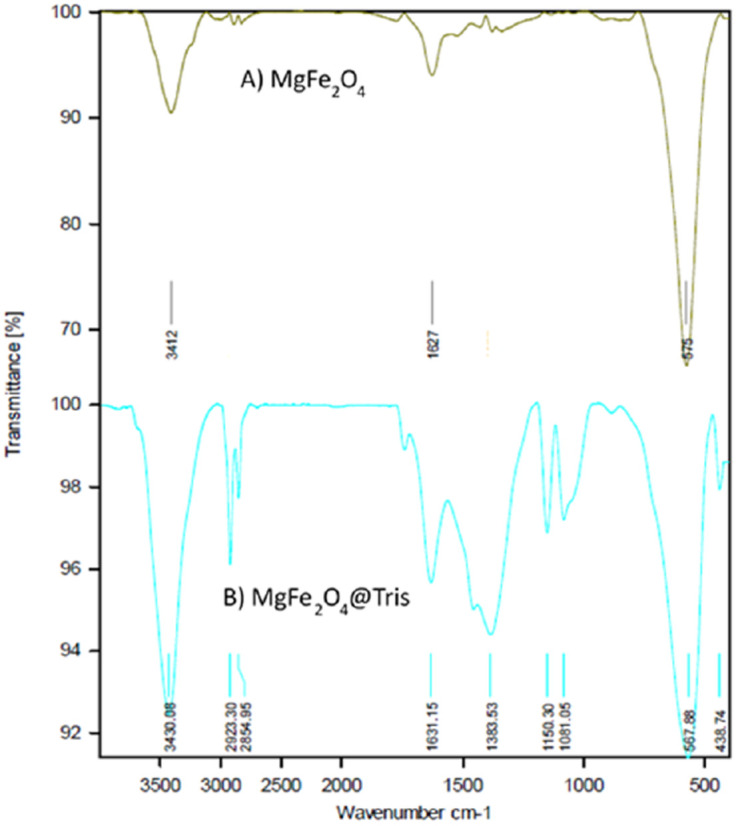
FT-IR spectra of (A) MgFe_2_O_4_ and (B) MgFe_2_O_4_@Tris MNPs.

The FT-IR spectra of MgFe_2_O_4_ and MgFe_2_O_4_@Tris MNPs are displayed in Fig. 1. The FT-IR spectrum of MgFe_2_O_4_ displayed bands at 3412 and 1627 cm^−1^ corresponding to the stretching and bending vibrations of the O–H bond. In addition, two peaks at 575 and 438 cm^−1^ correspond to Mg–O stretches (Fig. 1A). Peaks at 2855, 2923, and 1383 were observed in the FT-IR spectra of MgFe_2_O_4_@Tris due to stretching and bending vibrations in C–H aliphatic groups. The absorption peaks at 3430 and 1631 cm^−1^ are attributed to the stretching and bending vibrations of N–H groups. The vibrations at 1081 and 1150 cm^−1^ are related to C–C and C–N bonds (Fig. 1B).

#### 3.1.2. XRD analysis

**Fig. 2:**
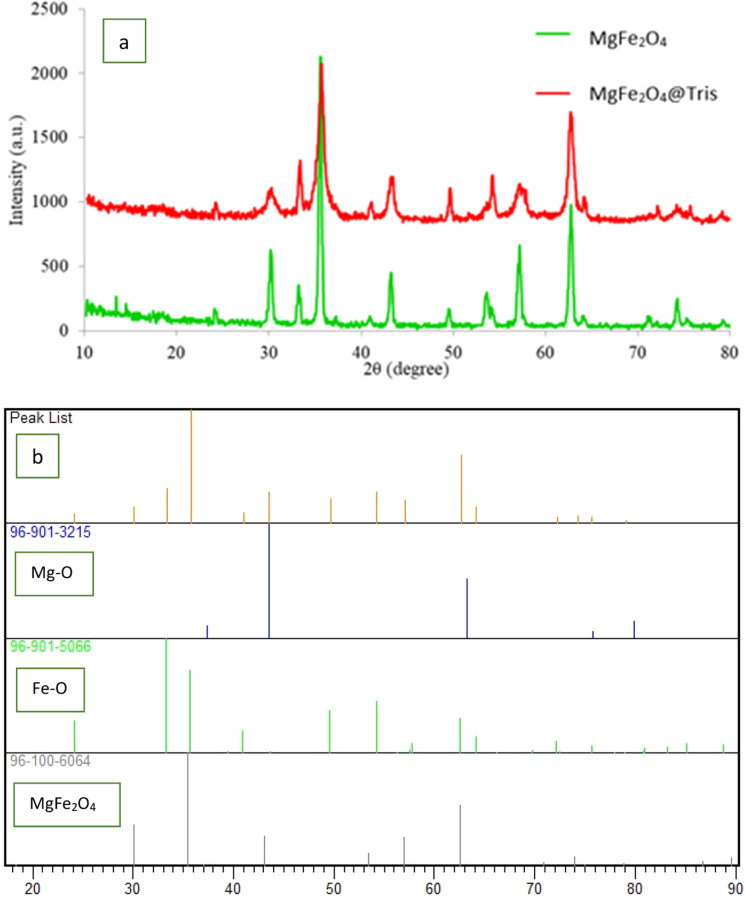
(a) XRD patterns of MgFe_2_O_4_ (green line) and MgFe_2_O_4_@Tris MNPs (red line). (b) Standard XRD patterns.

To recognize the formation of the magnetite crystal phase in the nanocatalyst, the synthesized samples were analyzed by X-ray diffraction (XRD). The XRD analyses of MgFe_2_O_4_ (green) and MgFe_2_O_4_@Tris (red) nanoparticles are shown in Fig. 2a. The X-ray diffraction planes at (111), (220), (311), (400), (422), (511), (440), (533), (622), and (444) confirm the formation of a cubic spinel structure. The locations of all peaks in the XRD analysis of MgFe_2_O_4_ were matched to the standard XRD analysis of MgFe_2_O_4_.^28^ These peaks showed that the structure of MgFe_2_O_4_ MNPs corresponds to the standard pattern with reference card numbers ICSD: 96-100-6064, ICSD: 96-901-3215 and ICSD: 96-901-5066 (Fig. 2b). The particle size determined from the XRD data was 59.09 nm, which was calculated using the Debye–Scherrer formula (*D* = *Kλ*/*β* cos *θ*).

#### 3.1.3. SEM analysis

**Fig. 3:**
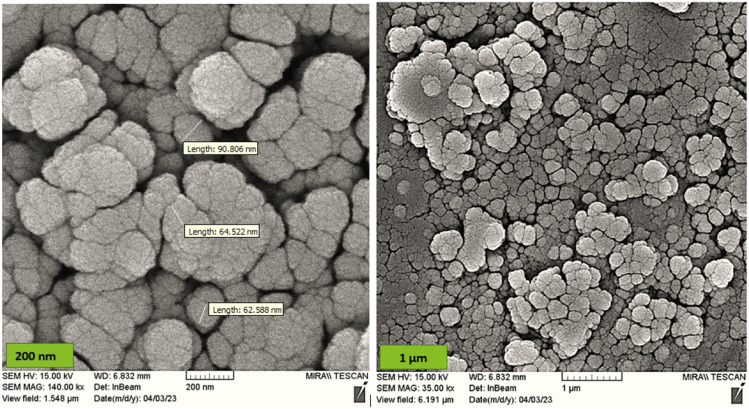
SEM images of MgFe_2_O_4_@Tris MNPs at different magnifications.

The morphology and particle size of the nano-solid magnesium ferrite were analyzed using scanning electron microscopy (SEM). As shown in Fig. 3, the images show a spherical structure with an average particle diameter of 65–90 nm and a uniform distribution and size.

#### 3.1.4. TEM analysis

**Fig. 4:**
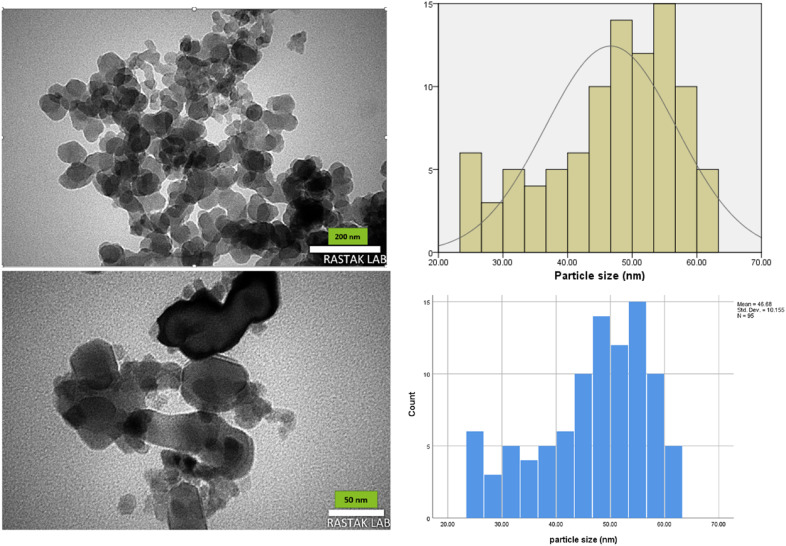
TEM images and histograms of MgFe_2_O_4_@Tris nanoparticles at different magnifications.

Transmission electron microscopy (TEM) analysis was used to examine the shape and size of the magnetic nanoparticles (Fig. 4). The images obtained confirm the formation of particles with a spherical morphology. Additionally, the particle size distribution histogram obtained from the TEM images revealed that the MgFe_2_O_4_@Tris nanoparticles have an average size of 46 nm.

It is shown from SEM, TEM, and XRD analyses that the catalyst has a nanometer-sized structure.

#### 3.1.5. EDX analysis

**Fig. 5:**
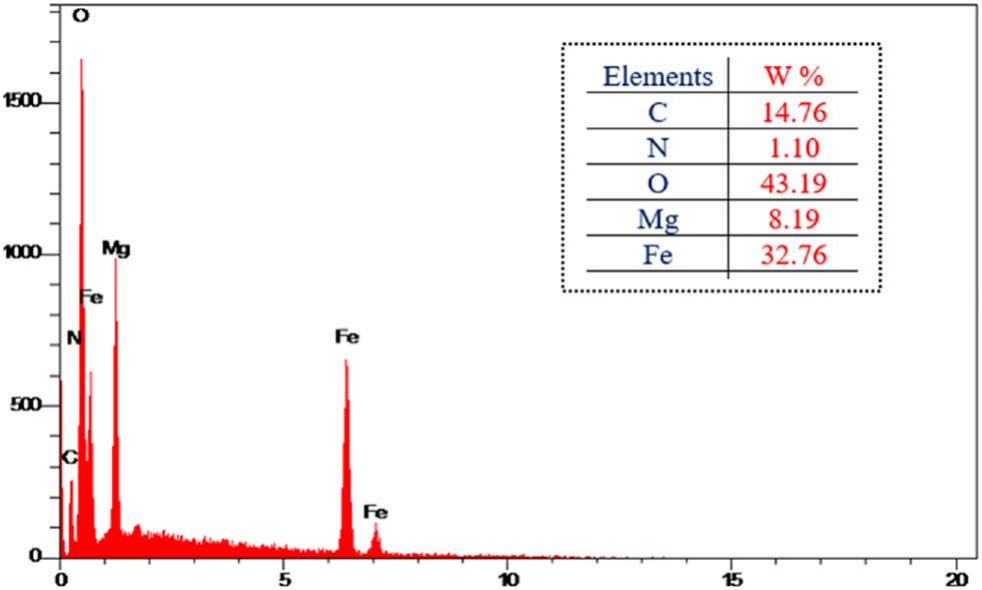
EDX spectrum of MgFe_2_O_4_@Tris MNPs.

**Fig. 6:**
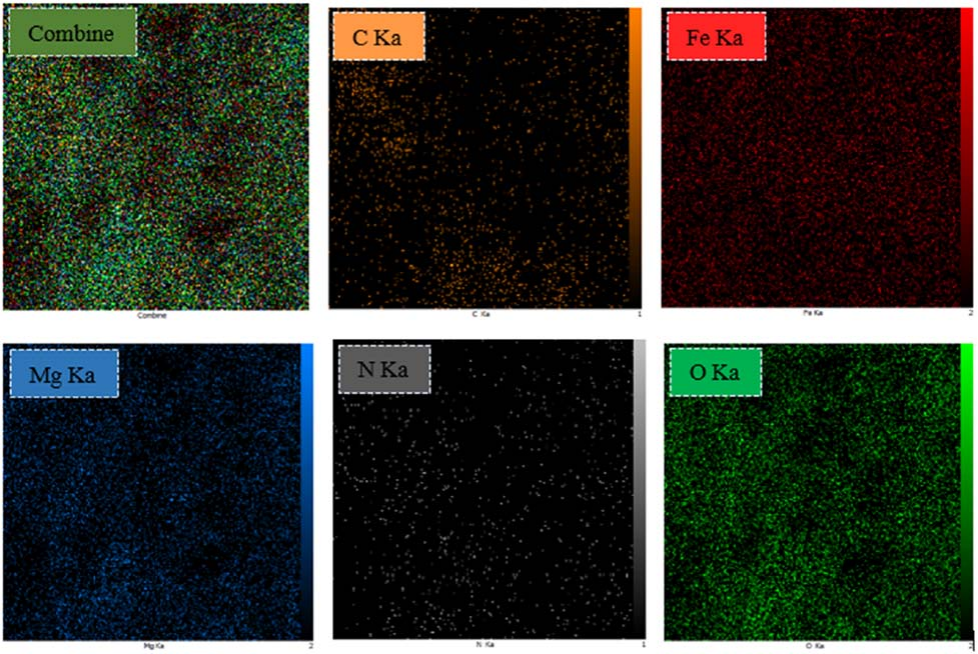
EDX mapping spectra of MgFe_2_O_4_@Tris MNPs.

Energy dispersive X-ray spectroscopy (EDX) is a powerful analytical tool for characterizing and determining elements present in catalyst components and the purity of the nanoparticles. The EDX spectrum of MgFe_2_O_4_@Tris is shown in Fig. 5, which confirms the elements Mg, Fe, O, N, and C in the nano-catalyst structure. Additionally, there are no peaks related to any impurities. It can be concluded that the nano-catalyst has been successfully produced. EDX mapping spectra of MgFe_2_O_4_@Tris confirm the synthesis of the catalyst. The elements were shown to be uniformly distributed in the nano-catalyst (Fig. 6).

#### 3.1.6. TGA analysis

**Fig. 7:**
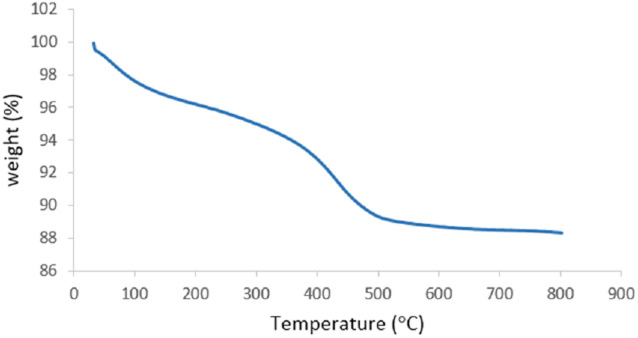
TGA thermogram of MgFe_2_O_4_@Tris MNPs.

The thermogravimetric analysis (TGA) diagram of the MgFe_2_O_4_@Tris nanoparticles is represented in Fig. 7. The TGA diagram of the MgFe_2_O_4_@Tris nanoparticles shows a small weight loss below 200 °C, corresponding to the evaporation of physically adsorbed solvents and OH groups on the nano-catalyst surface, and the next weight loss at temperatures above 200 °C is related to the removal of organic moieties on the MgFe_2_O_4_@Tris nanoparticles.

#### 3.1.7. VSM analysis

**Fig. 8:**
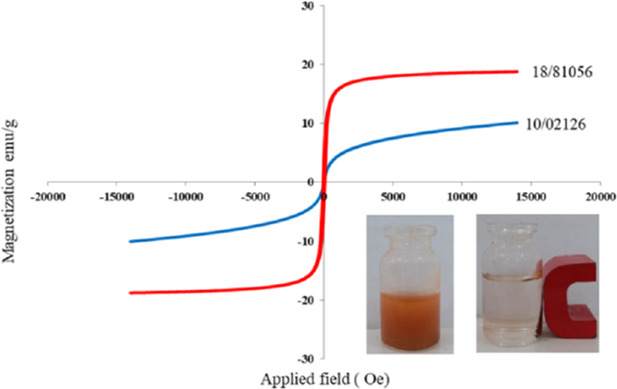
VSM diagram of MgFe_2_O_4_ (red line) and MgFe_2_O_4_@Tris MNPs (blue line).

The magnetization characteristics of the MgFe_2_O_4_ and MgFe_2_O_4_@Tris nanoparticles were analyzed using a vibrating sample magnetometer (VSM). The magnetization diagrams of MgFe_2_O_4_ (red line) and MgFe_2_O_4_@Tris (blue line) are shown in Fig. 8. The values of the saturation magnetization of the MgFe_2_O_4_ and MgFe_2_O_4_@Tris nanoparticles are 18 and 10 emu/g, respectively. The observed decrease in magnetic moment (MS) results from the addition of diamagnetic organic species to the surface of the MNPs, which confirms the presence of Tris on the surface of the MgFe_2_O_4_ nanoparticles. Meanwhile, the catalyst can be easily recycled from the reaction mixture using a simple magnetic field.

### 3.2. Catalytic studies

Following the successful synthesis and full characterization of MgFe_2_O_4_@Tris MNPs, we investigated the efficiency and activity of this nano-catalyst in the synthesis of pyrazolopyranopyrimidine and tetrahydrodipyrazolopyridine derivatives.

#### 3.2.1. Pyrazolopyranopyrimidine

**Scheme 3:**
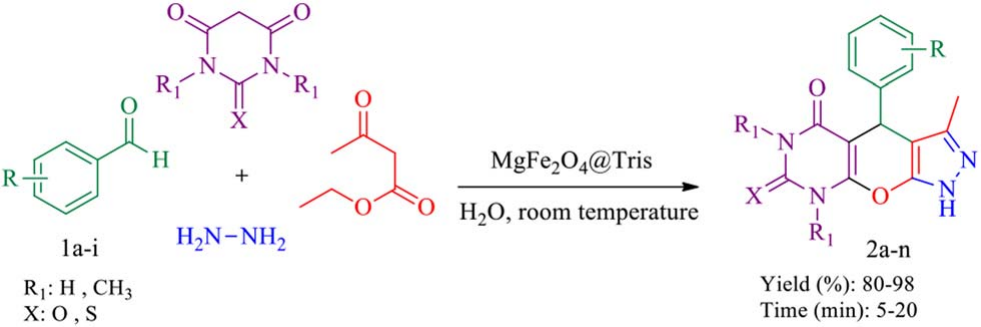
Synthesis of pyrazolopyranopyrimidine derivatives catalysed by MgFe_2_O_4_@Tris MNPs.

A four-component reaction between *para*-chlorobenzaldehyde, ethyl acetoacetate, hydrazine hydrate and barbituric acid was used as the sample reaction, and the effect of diverse conditions involving different temperatures, catalyst amounts, and solvents was examined. The results are summarised in Table 1. The best efficiency of product 2a is obtained when conducting the reaction in water at room temperature and in the presence of 10 mg of MgFe_2_O_4_@Tris MNPs (Table 1, entry 3). To verify the catalytic activity, we expanded the reaction to a range of aromatic aldehydes and barbituric acid under optimal reaction conditions (Scheme 3), and the results are reported in Table 2.

**Table 1:** Optimization of reaction conditions in the sample reaction^a^

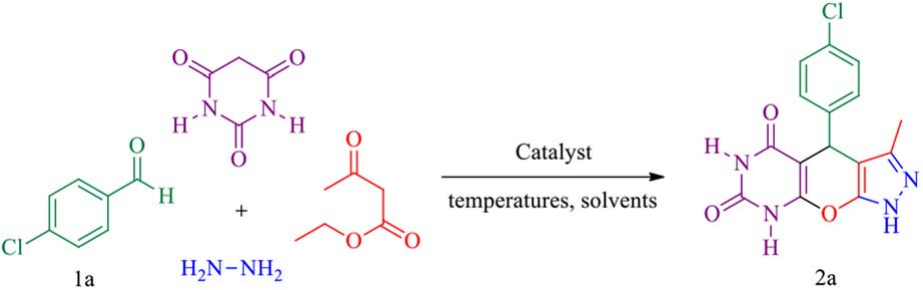					
Entry	Amount of catalyst (mg)	Solvent	Temp (°C)	Time (min)	Yield^b^ (%)
1	30	H_2_O	rt	15	98
2	20	H_2_O	rt	10	98
**3**	**10**	**H**_**2**_ **O**	**rt**	**10**	**98**
4	5	H_2_O	rt	10	90
5	10	H_2_O/EtOH	rt	10	97
6	10	EtOH	rt	20	85
7	10	DMF	rt	30	75
8	10	CH_3_CN	rt	50	65
9	10	DCM	rt	120	20
10	10	EtOAc	rt	60	75
11	10	*n*-Hexane	rt	120	Trace
12	10	H_2_O	40	10	98
13	10	H_2_O	60	10	98

a Reaction conditions: hydrazine hydrate (1 mmol), ethyl acetoacetate (1 mmol), *para*-chlorobenzaldehyde (1 mmol) and barbituric acid (1 mmol) in the presence of catalyst and solvent (2 mL).

b Isolated yield.

**Table 2:** Synthesis of pyrazolopyranopyrimidine derivatives catalysed by MgFe_2_O_4_@Tris MNPs

Entry	Aldehyde	R_1_	X	Product	Time (min)	Yield^a^ (%)	M.p. (°C) (ref.)
1	4-Cl C_6_H_5_	H	O	2a	10	98	218–220 (ref. 15)
2	C_6_H_5_	H	O	2b	7	98	215–218 (ref. 15)
3	4-OMe C_6_H_5_	H	O	2c	15	97	226–228 (ref. 15)
4	2-OH C_6_H_5_	H	O	2d	10	97	264–268 (ref. 17)
5	2-OMe C_6_H_5_	H	O	2e	15	92	228–230 (ref. 15)
6	3-OH C_6_H_5_	H	O	2f	10	94	278–280 (ref. 30)
7	3-NO_2_ C_6_H_5_	H	O	2g	20	80	266–268 (ref. 17)
8	4-Br C_6_H_5_	H	O	2h	20	95	210–212 (ref. 30)
9	4-Cl C_6_H_5_	H	S	2i	5	98	224–226 (ref. 35)
10	4-OMe C_6_H_5_	H	S	2j	10	92	224–225 (ref. 15)
11	C_6_H_5_	H	S	2k	5	94	219–220 (ref. 15)
12	2-OH C_6_H_5_	H	S	2l	5	95	267–270 (ref. 35)
13	2-OMe C_6_H_5_	H	S	2m	5	97	222–225 (ref. 15)
14	4-Cl C_6_H_5_	CH_3_	O	2n	10	98	200–202 (ref. 15)

a Isolated yield.

**Scheme 4:**
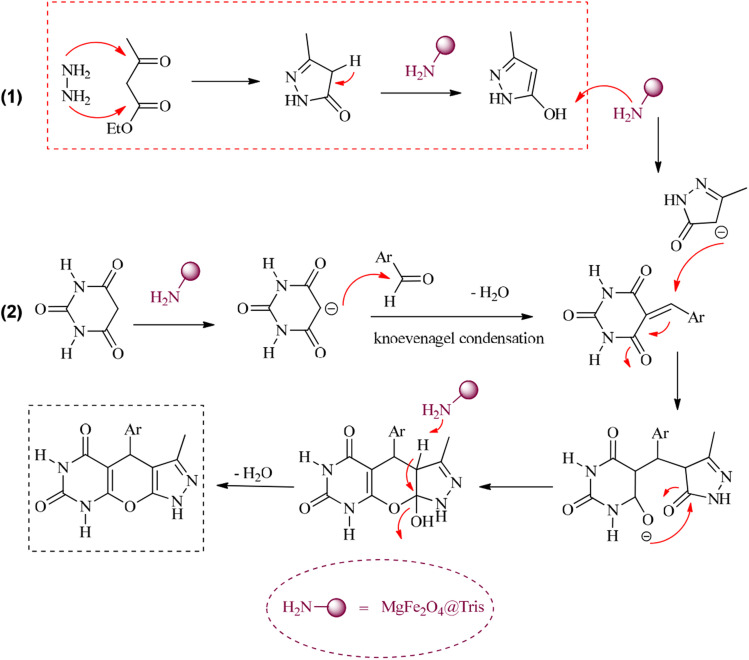
Proposed mechanism for the synthesis of pyrazolopyranopyrimidines using MgFe_2_O_4_@Tris MNPs.

A proposed mechanism for the synthesis of pyrazolopyranopyrimidines using MgFe_2_O_4_@Tris MNPs as the catalyst is shown in Scheme 4.^15^

#### 3.2.2. Tetrahydrodipyrazolopyridine

**Scheme 5:**
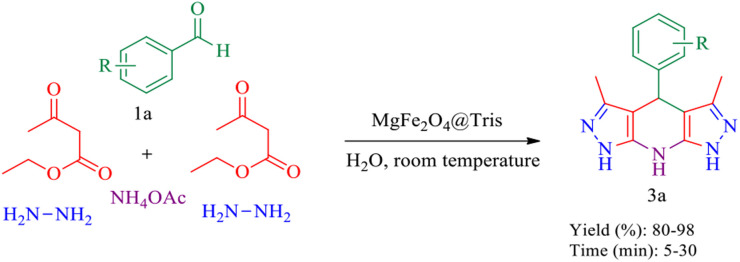
Synthesis of tetrahydrodipyrazolopyridine derivatives catalysed by MgFe_2_O_4_@Tris MNPs.

**Scheme 6:**
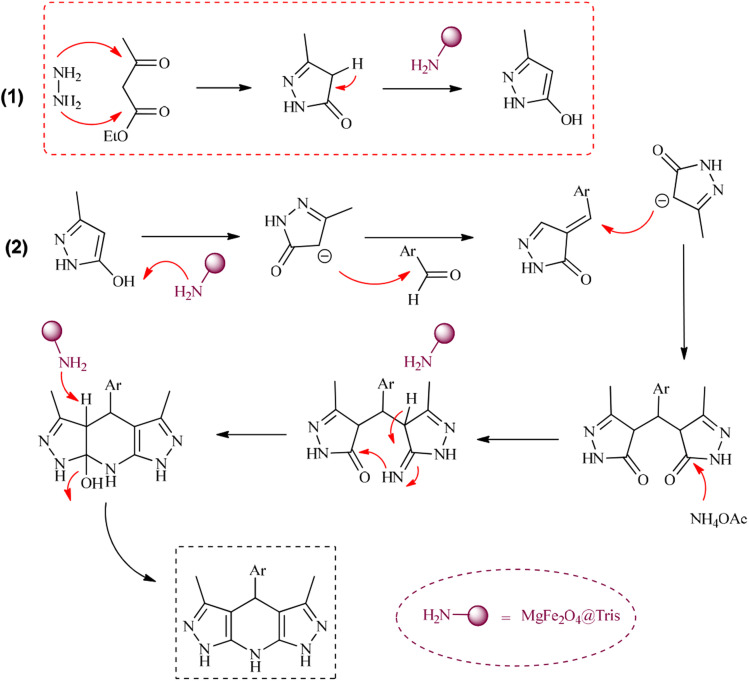
Proposed mechanism for the synthesis of tetrahydrodipyrazolopyridine using MgFe_2_O_4_@Tris MNPs.

We used MgFe_2_O_4_@Tris MNPs to prepare tetrahydrodipyrazolopyridine derivatives (Scheme 5). To optimize the amount of catalyst, solvent, and reaction temperature, the reaction of ethyl acetoacetate, hydrazine hydrate, *para*-chlorobenzaldehyde, and ammonium acetate was selected as the model reaction (Table 3). The best efficiency of product 3a is achieved when the reaction is carried out in H_2_O as the solvent at room temperature and in the presence of 15 mg of MgFe_2_O_4_@Tris MNPs (Table 3, entry 3). Subsequently, a vast range of tetrahydrodipyrazolopyridine derivatives was synthesized with success under optimal conditions with excellent yields and short reaction times, and the results of these reactions are presented in Table 4. The suggested mechanism for the production of tetrahydrodipyrazolopyridine using MgFe_2_O_4_@Tris MNPs as a catalyst is shown in Scheme 6.^38^

**Table 3:** Optimization of reaction conditions in the sample reaction^a^

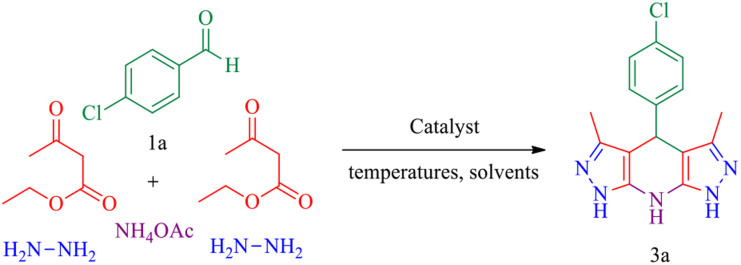					
Entry	Amount of catalyst (mg)	Solvent	Temp (°C)	Time (min)	Yield^b^ (%)
1	30	H_2_O	rt	10	98
2	20	H_2_O	rt	10	98
**3**	15	**H**_**2**_ **O**	**rt**	**10**	**98**
4	10	H_2_O	rt	15	90
5	15	H_2_O/EtOH	rt	10	95
6	15	EtOH	rt	45	50
7	15	DMF	rt	30	95
8	15	CH_3_CN	rt	120	20
9	15	EtOAc	rt	120	Trace
10	15	*n*-Hexane	rt	120	Trace
11	15	H_2_O	40	10	98
12	15	H_2_O	60	10	98

a Reaction conditions: hydrazine hydrate (2 mmol), ethyl acetoacetate (2 mmol), *para*-chlorobenzaldehyde (1 mmol) and ammonium acetate (1.5 mmol) in the presence of catalyst and solvent (2 mL).

b Isolated yield.

**Table 4:** Synthesis of tetrahydrodipyrazolopyridine derivatives catalysed by MgFe_2_O_4_@Tris MNPs

Entry	Aldehyde	Product	Time (min)	Yield^a^ (%)	M.p. (°C) (ref.)
1	4-Cl C_6_H_5_	3a	10	98	252–254 (ref. 16)
2	C_6_H_5_	3b	5	98	241–243 (ref. 16)
3	4-OMe C_6_H_5_	3c	20	90	187–190 (ref. 16)
4	2-OMe C_6_H_5_	3d	15	93	180–182 (ref. 37)
5	2-NO_2_ C_6_H_5_	3e	20	88	189–190 (ref. 16)
6	3-NO_2_ C_6_H_5_	3f	20	85	284–286 (ref. 16)
7	4-Br C_6_H_5_	3g	25	91	169–170 (ref. 16)
8	4-OH C_6_H_5_	3h	20	92	269–270 (ref. 36)
9	2-Cl C_6_H_5_	3i	15	90	165–167 (ref. 16)
10	4-NO_2_ C_6_H_5_	3j	20	80	298–300 (ref. 16)
11	3-Br C_6_H_5_	3k	30	91	247–248 (ref. 16)
12	(CH_3_)_2_ N C_6_H_5_	3l	30	90	240–243 (ref. 16)
13	Terephthalaldehyde	3m	10	96	>300 (ref. 37)

a Isolated yield.

#### 3.2.3. Comparison with other catalysts

To demonstrate the superiority and high efficiency of the nano-catalyst synthesized in this research, the results obtained for the synthesis of pyrazolopyranopyrimidines and tetrahydrodipyrazolopyridines using MgFe_2_O_4_@Tris MNPs were compared with the catalysts reported in previous studies. As shown in Table 5, this nano-catalyst has advantages such as high catalytic activity, low reaction time and excellent product yields.

**Table 5:** Comparison of the catalytic activity of MgFe_2_O_4_@Tris MNPs with other catalysts in the synthesis of products 2a (entries 1–5) and 3a (entries 6–10)

Entry	Catalyst	Conditions	Time (min)	Yield (%)	Ref.
1	[BNPs-Caff]HSO_4_	H_2_O, 50 °C	45	92	29
2	*β*-Cyclodextrin	H_2_O, 40 kHz, 50 °C	30	90	30
3	[MerDABCO-SO_3_H]Cl	H_2_O, 80 °C	15	94	17
4	H_3_[PMo_7_W_5_O_40_]·24H_2_O	Solvent free, 80 °C	40	94	31
**5**	**MgFe**_**2**_ **O**_**4**_ **@Tris**	**H**_**2**_ **O, rt**	**10**	**98**	**This work**
6	Pseudopolymeric magnetic nanoparticles	EtOH, rt	60	63	16
7	HANCD@urease	H_2_O, 70 °C	70	95	32
8	Nano-CdZr_4_(PO_4_)_6_	EtOH, reflux	40	92	33
9	Nano-ovalbumin	H_2_O, 55 °C	35	94	34
**10**	**MgFe**_**2**_ **O**_**4**_ **@Tris**	**H**_**2**_ **O, rt**	**10**	**98**	**This work**

#### 3.2.4. Catalyst recovery and reuse

**Fig. 9:**
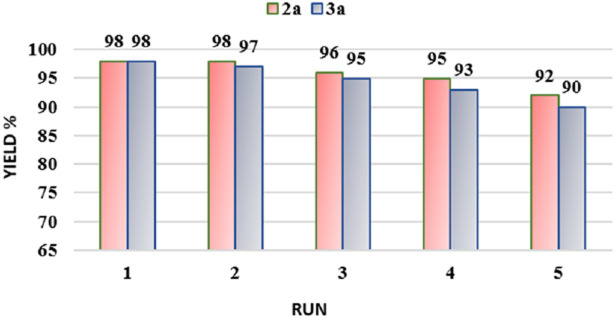
Possibility of reusing MgFe_2_O_4_@Tris MNPs in the synthesis of products 2a and 3a.

**Fig. 10:**
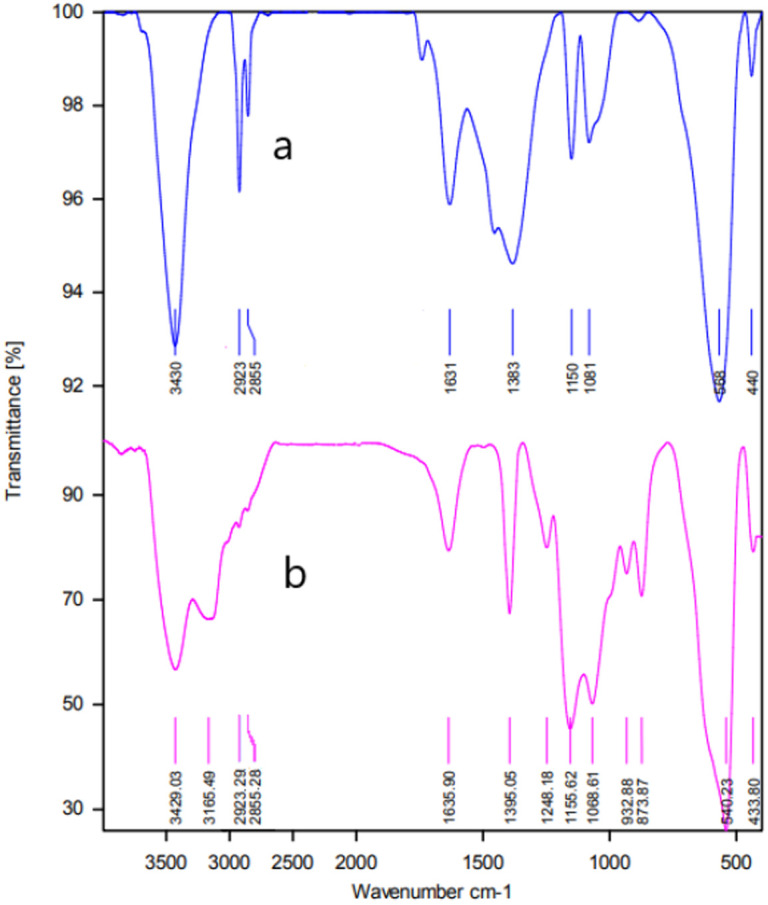
FT-IR spectrum of fresh MgFe_2_O_4_@Tris (a) and MgFe_2_O_4_@Tris after recovery (b).

**Fig. 11:**
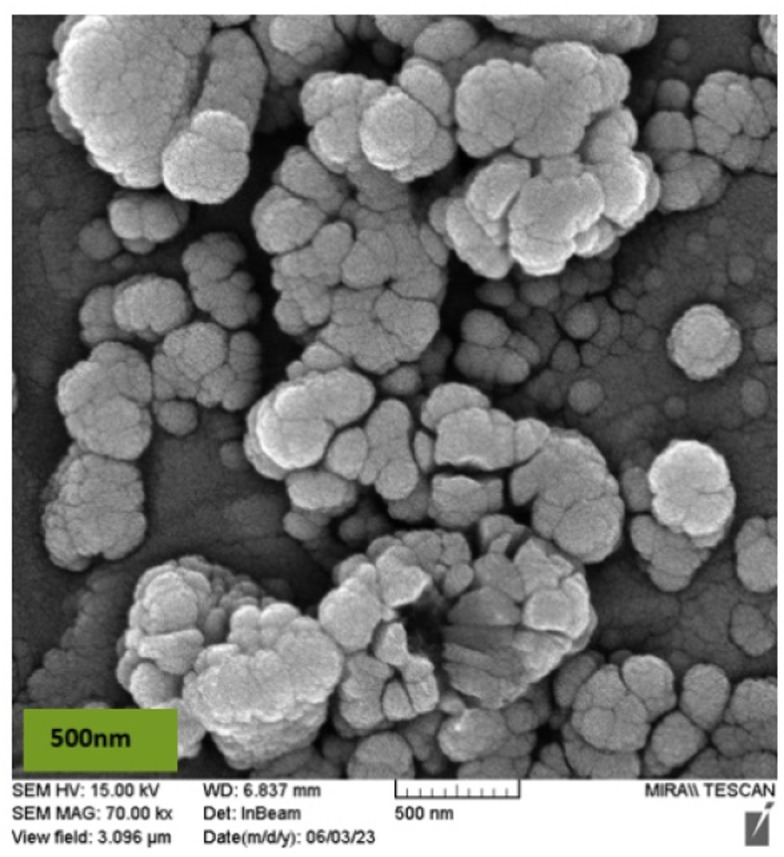
SEM images of MgFe_2_O_4_@Tris MNPs after recovery.

One of the important features of the catalyst synthesized in this study is its magnetism. This feature simplifies catalyst recovery and separation. The possibility of reusing the MgFe_2_O_4_@Tris nano-catalyst in the synthesis of products 2a and 3a (as model reactions) was considered. The results are presented in Fig. 9. In summary, the catalytic activity was tested over 5 cycles, and similar activities were displayed, without any notable loss of the original catalytic activity. Additionally, the FT-IR spectra and SEM images of MgFe_2_O_4_@Tris before and after recovery (Fig. 10 and 11) exhibited good similarity due to the stability of the catalyst.

## 4. Conclusions

We introduced an innovative magnetic heterogeneous nano-catalyst, made from MgFe_2_O_4_ nanoparticles and tris(hydroxymethyl)aminomethane using a co-precipitation method. Various techniques were used for the full characterization of the synthesized nanocatalysts. The catalytic activity of this nanocatalyst for the synthesis of pyrazolopyranopyrimidine and tetrahydrodipyrazolopyridine derivatives was evaluated. A key feature of this protocol was a high product yield, and this catalyst can simply be separated from the reaction mixture using an external magnet and reused for multiple cycles without appreciable loss of its original catalytic activity. To summarize this study, this method can serve as a powerful strategy for the synthesis of important active molecules.

## Author contributions

All authors contributed to data analysis, supervised the project, drafted and revised the paper and agreed to be responsible for all aspects of this work.

## Conflicts of interest

The authors declare no conflicts of interest.

## Supplementary Material

RA-014-D3RA07934A-s001
