# Supplementary material for: MgFe2O4@Tris magnetic nanoparticles: an effective and powerful catalyst for one-pot synthesis of pyrazolopyranopyrimidine and tetrahydrodipyrazolopyridine derivatives
Source: RSC Adv. 2024 Feb 15;14(9):6006–15. doi: 10.1039/d3ra07934a (PMC10868241; doi:10.1039/d3ra07934a)
Supplement: RA-014-D3RA07934A-s001 [file RA-014-D3RA07934A-s001.pdf]

Electronic Supplementary Material (ESI) for RSC Advances.
This journal is © The Royal Society of Chemistry 2024

Supporting Information

$MgFe_2O_4$@Tris magnetic nanoparticles: an effective and powerful catalyst for one-pot synthesis of pyrazolopyranopyrimidine and tetrahydrodipyrazolopyridine derivatives

Zahra Ramezaninejad[1] and Lotfi Shiri *[1]

E-mail: lshiri47@gmail.com and l.shiri@ilam.ac.ir
[1] Department of Chemistry, Faculty of Sciences, Ilam University, P.O. Box 69315516, Ilam, Iran

Abstract

Magnesium (Mg) as a metal has wide applications, but its use in chemical reactions is rarely reported. Currently, magnesium catalytic processes are being developed to synthesize basic chemical compounds. Therefore, an effective and recyclable nano-catalyst was synthesized using $MgFe_2O_4$@Tris in this study. The structure of the $MgFe_2O_4$@Tris was characterized by various techniques including Fourier-transform infrared (FT-IR), scanning electron microscopy (SEM), transmission electron microscopy (TEM), energy dispersive X-ray (EDX), thermogravimetric analysis (TGA), X-ray diffraction (XRD), and vibrating sample magnetometer (VSM) techniques. Finally, the catalytic activity of this nano-catalyst was evaluated for the synthesis of pyrazolopyranopyrimidine and tetrahydrodipyrazolopyridine derivatives. Among the advantages of this catalyst are its high catalytic activity, high yields, use of environmentally friendly solvents, easy magnetic separation, and the possibility of reusing the catalyst.

General information about the equipment, solvents and raw materials used

All reactants used in this research were purchased from Merck, Fluka, or Sigma-Aldrich Chemical Companies. The melting points were defined by Barnstead Electrothermal 9100 in capillary tubes. The Infrared (IR) spectra of samples were recorded in KBr pellets using a VRTEX 70 spectrophotometer (Bruker, Germany). ^{13}C and ^{1}H NMR spectra (in Hertz) were obtained by Bruker DRX-250 AVANCE instrument in DMSO-d_6 as the solvent and TMS as the internal standard. Energy-dispersive X-ray spectroscopy (EDX) and scanning electron microscopy (SEM) were accomplished and utilized a Czech TESCAN instrument. Thermogravimetric analysis (TGA) was performed using a thermogravimetric analyzer (PerkinElmer-STA6000, USA), and magnetic measurements of the nanocatalyst were obtained using a vibrating sample magnetometer (VSM; MDKB). X-ray diffraction (XRD) was collected using a Holland Philips PW1730 and TEM of the MNPs was recorded with a Philips-EM 208S TEM.

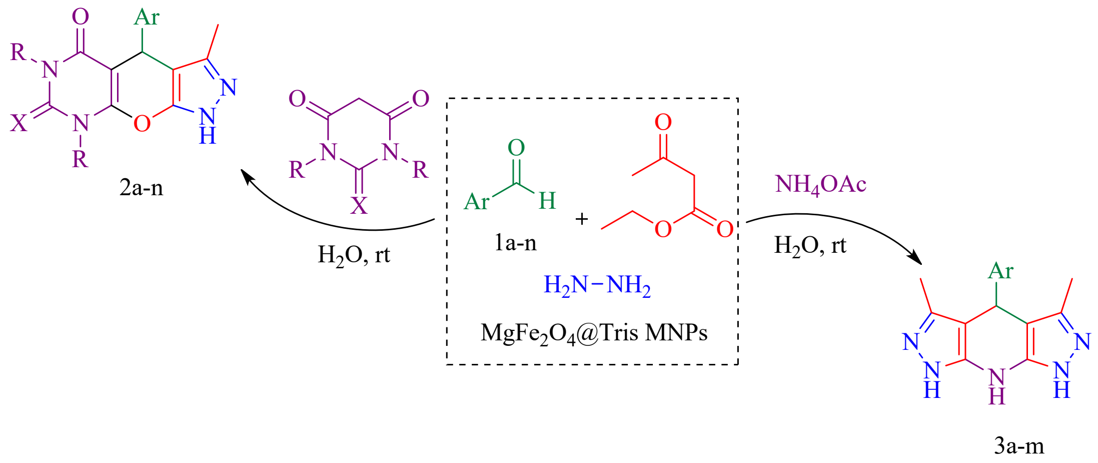

Scheme S1. Synthesis of pyrazolopyranopyrimidines and tetrahydrodipyrazolopyridine using $MgFe_2O_4$@Tris MNPs

Data on the physical properties of pyrazolopyranopyrimidines

3-Methyl-4-(4-chlorophenyl)-6,8-dihydropyrazolo[4',3':5,6]pyrano[2,3-d]pyrimidine-5,7(1H,4H)-dione (2a) (Table 2, Entry 1)

White solid; Yield: 98%; M.p. 218-220°C.

3-Methyl-4-phenyl-6,8-dihydropyrazolo[4',3':5,6]pyrano[2,3-d]- pyrimidine-5,7(1H,4H)-dione (2b) (Table 2, Entry 2)

White solid; Yield: 98%; M.p. 215-218°C.

3-Methyl-4-(4-methoxyphenyl)-1,4-dihydropyrazolo[4',3':5,6]pyrano[2,3-d]pyrimidine-5,7 (6H,8H)-diones (2c) (Table 2, Entry 3)

Yellow solid; Yield: 97%; M.p. 226-228°C.

3-Methyl-4-(2-methoxyphenyl)-1,4-dihydropyrazolo[4',3':5,6]pyrano[2,3-d]pyrimidine-5,7 (6H,8H)-diones (2e) (Table 2, Entry 5)

Pale yellow solid; Yield: 92%; M.p. 228-230°C.

3-Methyl-4-(3-hydroxyphenyl)-1,4-dihydropyrazolo[4',3':5,6]pyrano[2,3-d]pyrimidine-5,7 (6H,8H)-diones (2f) (Table 2, Entry 6)

Pale yellow solid; Yield: 94%; M.p. 278-280°C.

3-Methyl-4-(4-bromophenyl)-1,4-dihydropyrazolo[4',3':5,6]pyrano[2,3-d]pyrimidine-5,7 (6H,8H)-diones (2h) (Table 2, Entry 8)

White solid; Yield: 95%; M.p. 210-212°C.

4-(4-Methoxyphenyl)-3-methyl-7-thioxo-4,6,7,8-tetrahydropyrazolo[5,6]pyrano[2,3-d]pyrimidine-5(1H)-one (2j) (Table 2, Entry 10)

yellow solid; Yield: 92%; M.p. 224-225°C.

4-phenyl-3-methyl-7-thioxo-4,6,7,8-tetrahydropyrazolo[5,6]pyrano[2,3-d]pyrimidine-5(1H)-one (2k) (Table 2, Entry 11)

yellow solid; Yield: 94%; M.p. 219-220°C.

4-(2-hydroxyphenyl)-3-methyl-7-thioxo-4,6,7,8-tetrahydropyrazolo[5,6]pyrano[2,3-d]pyrimidine-5(1H)-one (2l) (Table 2, Entry 12)

yellow solid; Yield: 95%; M.p. 267-270°C.

4-(4-Chlorophenyl)-3,6,8-trimethyl-6,8-dihydropyrazolo(4',3':5,6)pyrano(2,3-d)pyrimidine-5,7(1H,4H)-dione (2n) (Table 2, Entry 14)

White solid; Yield: 98%; M.p. 200-202°C.

4-(2-Methoxyphenyl)-3-methyl-7-thioxo-4,6,7,8-tetrahydropyrazolo[5,6]pyrano[2,3-d]pyrimidine-5(1H)-one (2m) (Table 2, Entry 13)

yellow solid; Yield: 97%; M.p. 222-225°C; IR (KBr): 3468, 3130, 2891, 1609, 1544, 1391, 1287, 1242 cm^{-1}; ^1H NMR (250 MHz, DMSO-d6) δ (ppm): 13.5 (br, 1H), 11.42 (s, 1H), 7.31 (d, J = 7.6 Hz, 1H), 7.10 (t, J = 7.9 Hz, 1H), 6.80 (dd, J

= 12.4, 7.4 Hz, 2H), 5.63 (s, 1H), 3.71 (s, 1H), 3.64 (s, 3H), 3.42 (q, J = 7.3 Hz, 1H), 2.26 (s, 3H), 1.03 (t, J = 7.2 Hz, 1H); ^{13}C NMR (63 MHz, DMSO-d6) δ (ppm): 173.18, 163.75, 159.89, 157.13, 144.36, 130.32, 128.85, 127.56, 120.00, 111.17, 106.09, 95.32, 56.50, 55.76, 26.53, 10.60.

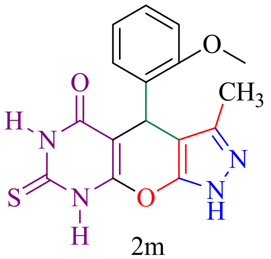

2m

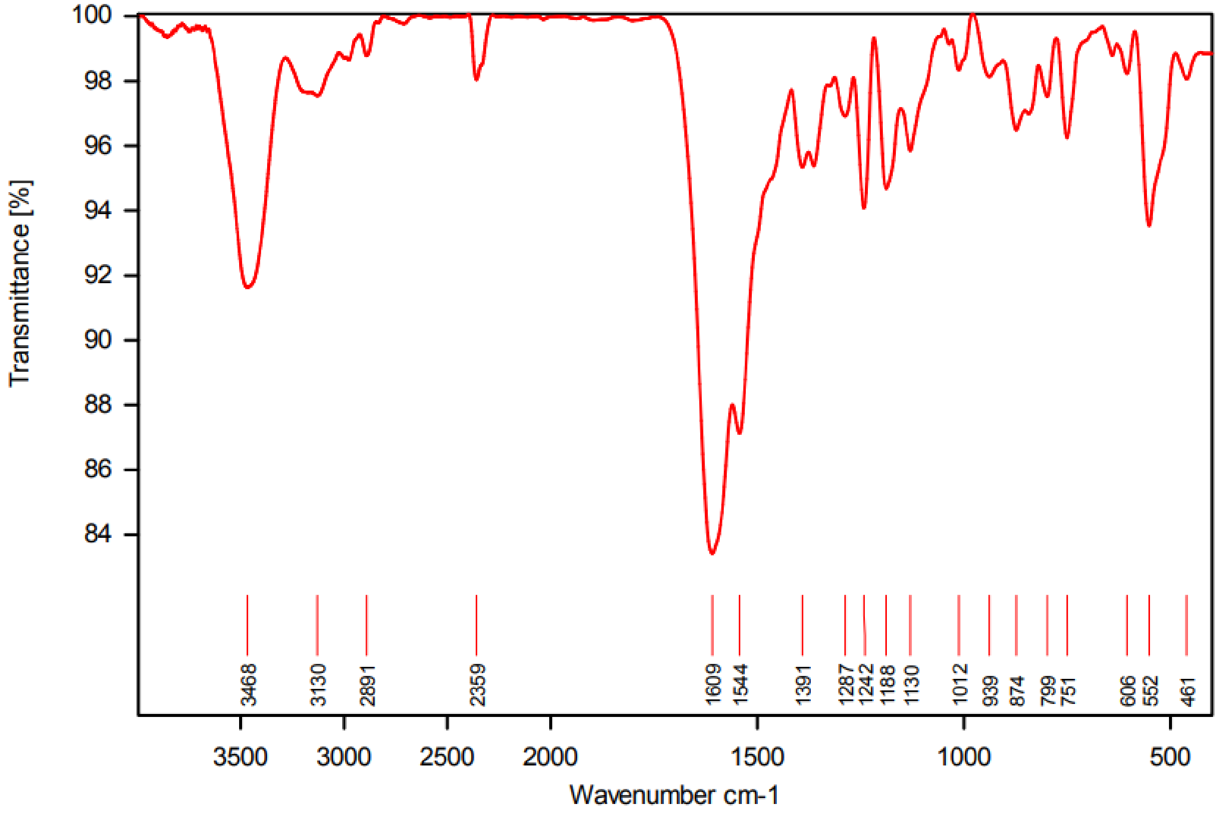

Figure S1. The FT-IR spectrum of 4-(2-Methoxyphenyl)-3-methyl-7-thioxo-4,6,7,8- tetrahydropyrazolo[5,6] pyrano[2,3-d]pyrimidine-5(1H)-one (2m) (Table 2, Entry 13)

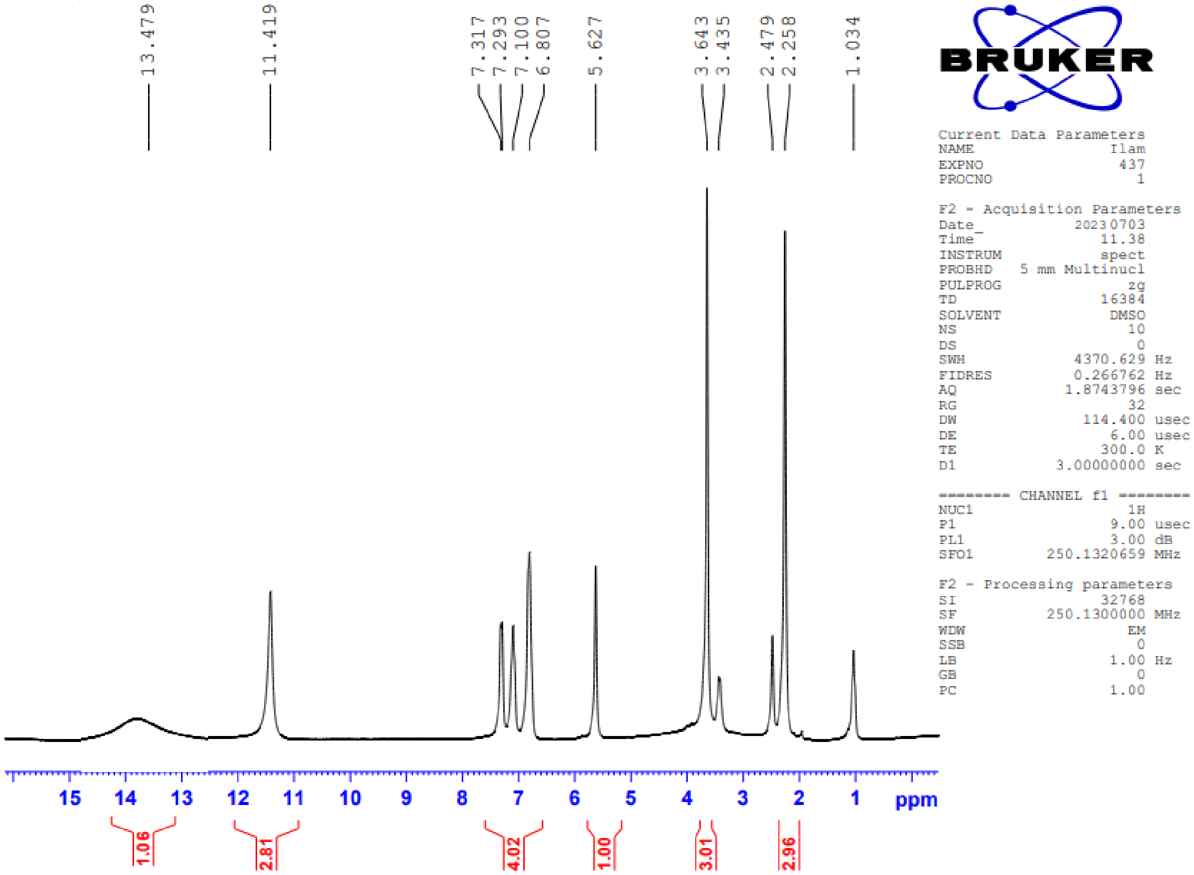

Figure S2. The ^{1}H NMR spectrum of 4-(2-Methoxyphenyl)-3-methyl-7-thioxo-4,6,7,8- tetrahydropyrazolo[5,6] pyrano[2,3-d]pyrimidine-5(1H)-one (2m) (Table 2, Entry 13)

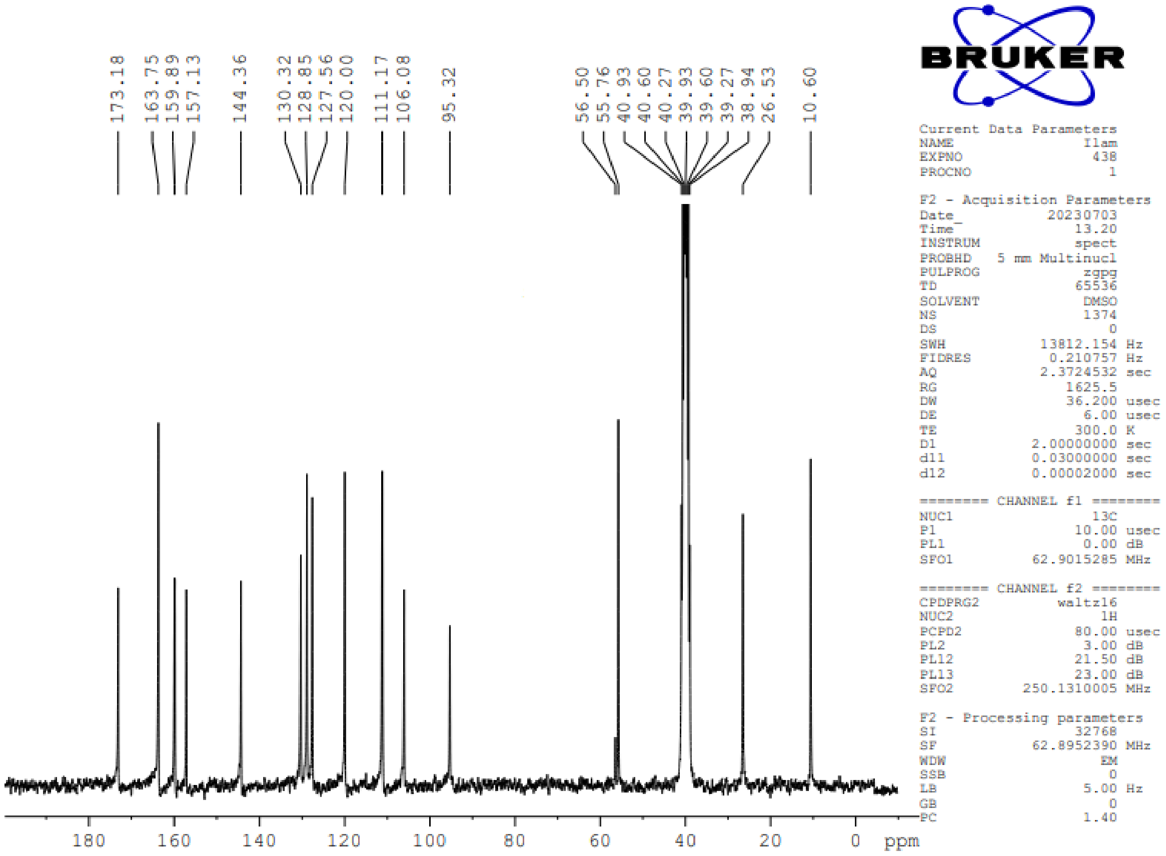

Figure S3. The ^{13}C NMR spectrum of 4-(2-Methoxyphenyl)-3-methyl-7-thioxo-4,6,7,8- tetrahydropyrazolo[5,6] pyrano[2,3-d]pyrimidine-5(1H)-one (2m) (Table 2, Entry 13)

3-Methyl-4-(2-hydroxyphenyl)-1,4-dihydropyrazolo[4',3':5,6]pyrano[2,3-d]pyrimidine-5,7(6H,8H)-diones (2d) (Table 2, Entry 4)

yellow solid; Yield: 97%; M.p. 264-268°C; ^{1}H NMR (250 MHz, DMSO-d6) δ (ppm): 13.3 (br, 1H), 10.08 (s, 3H), 7.25 (s, 2H), 6.90 (s, 2H), 6.62 (s, 4H), 5.54 (s, 1H), 4.50 (s, 1H), 4.11 (s, 1H), 2.19 (s, 6H), 1.26 (s, 2H); ^{13}C NMR (63 MHz, DMSO-d6) δ (ppm): 165.3, 160.99, 155.09, 151.00, 144.1, 132.9, 132.0, 129.24, 126.96, 118.51, 115.22, 106.1, 90.91, 81, 26. 18.92, 10.79.

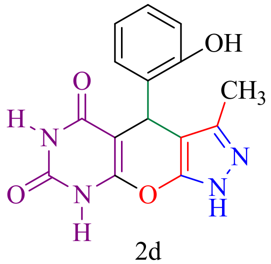

2d

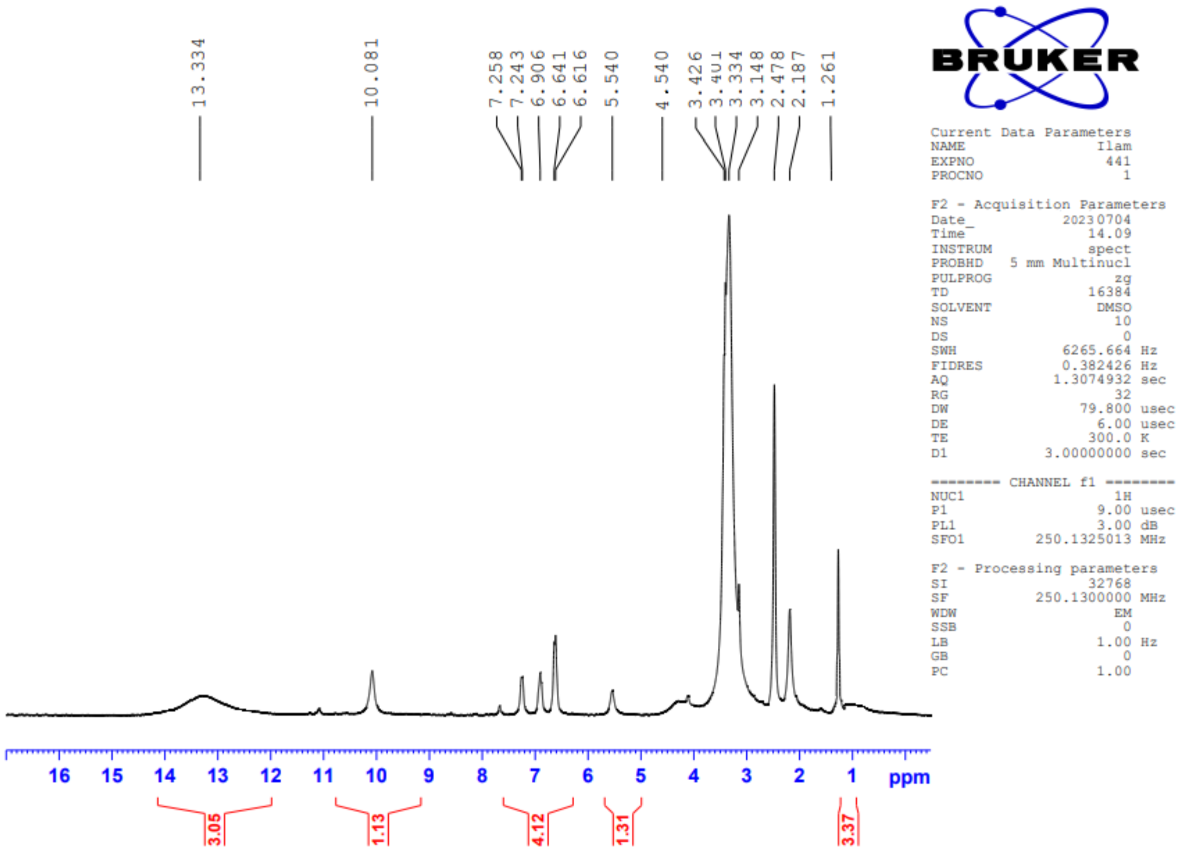

Figure S4. The ^{1}H NMR spectrum of 3-Methyl-4-(2-hydroxyphenyl)-1,4-dihydropyrazolo[4',3':5,6]pyrano[2,3-d]pyrimidine-5,7 (6H,8H)-diones (2f) (Table 2, Entry 4)

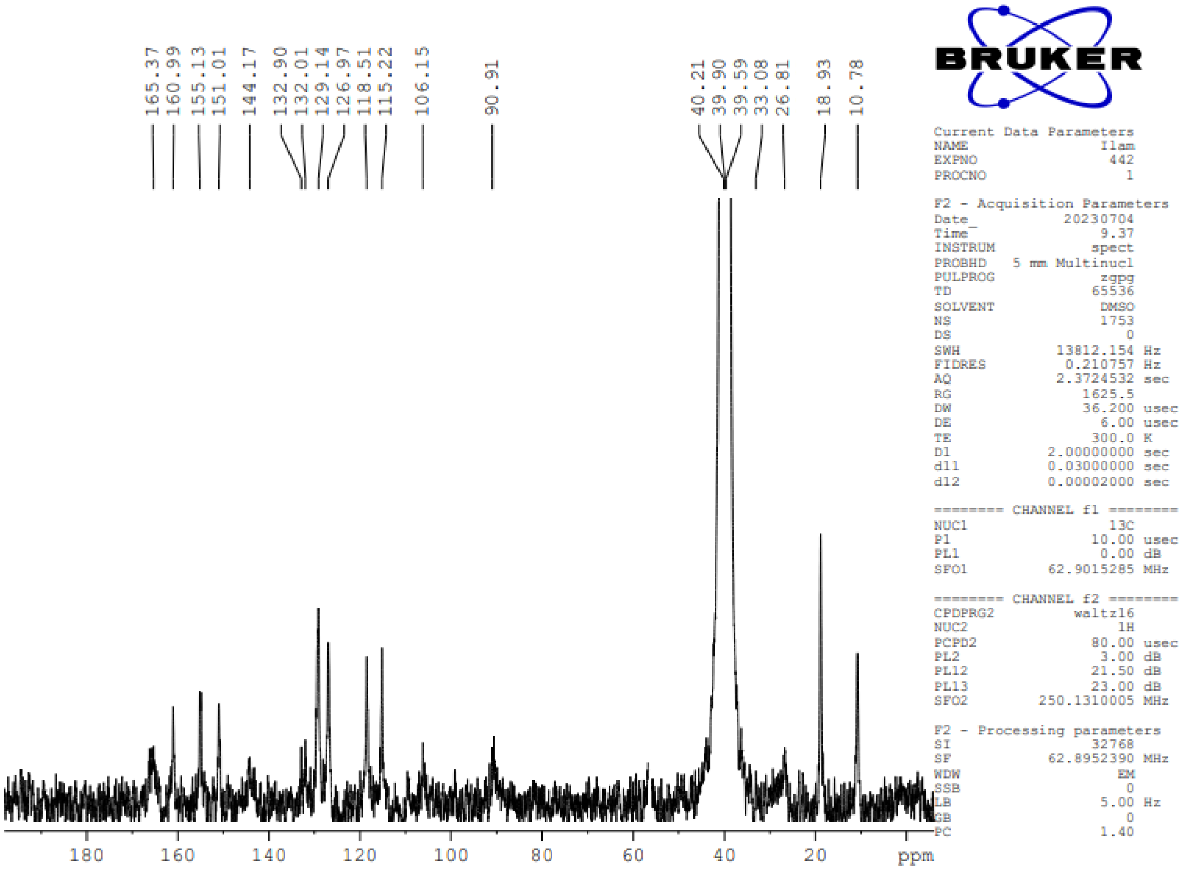

Figure S5. The ^{13}C NMR spectrum of 3-Methyl-4-(2-hydroxyphenyl)-1,4-dihydropyrazolo[4’,3’:5,6]pyrano[2,3-d]pyrimidine-5,7 (6H,8H)-diones (2d) (Table 2, Entry 4)

4-(4-chlorophenyl)-3-methyl-7-thioxo-4,6,7,8-tetrahydropyrazolo[5,6]pyrano[2,3-d]pyrimidine-5(1H)-one (2i) (Table 2, Entry 9)

White solid; Yield: 98%; M.p. 224-226°C; IR (KBr): 3418, 2873, 1597, 1533, 1351, 1308, 1190 cm^{-1}; ^{1}H NMR (250 MHz, DMSO-d6) δ (ppm): 11.49 (s, 2H), 7.24 (s, 2H), 7.03 (s, 2H), 5.39 (s, 1H), 2.23 (s, 3H); ^{13}C NMR (63 MHz, DMSO-d6) δ (ppm): 173.61, 163.73, 159.51, 144.21, 141.34, 130.57, 129.00, 128.27, 105.50, 96.23, 30.67, 10.38.

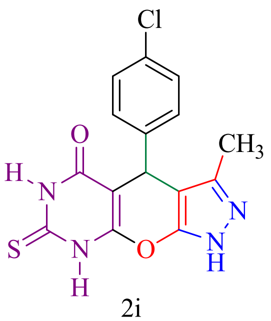

2i

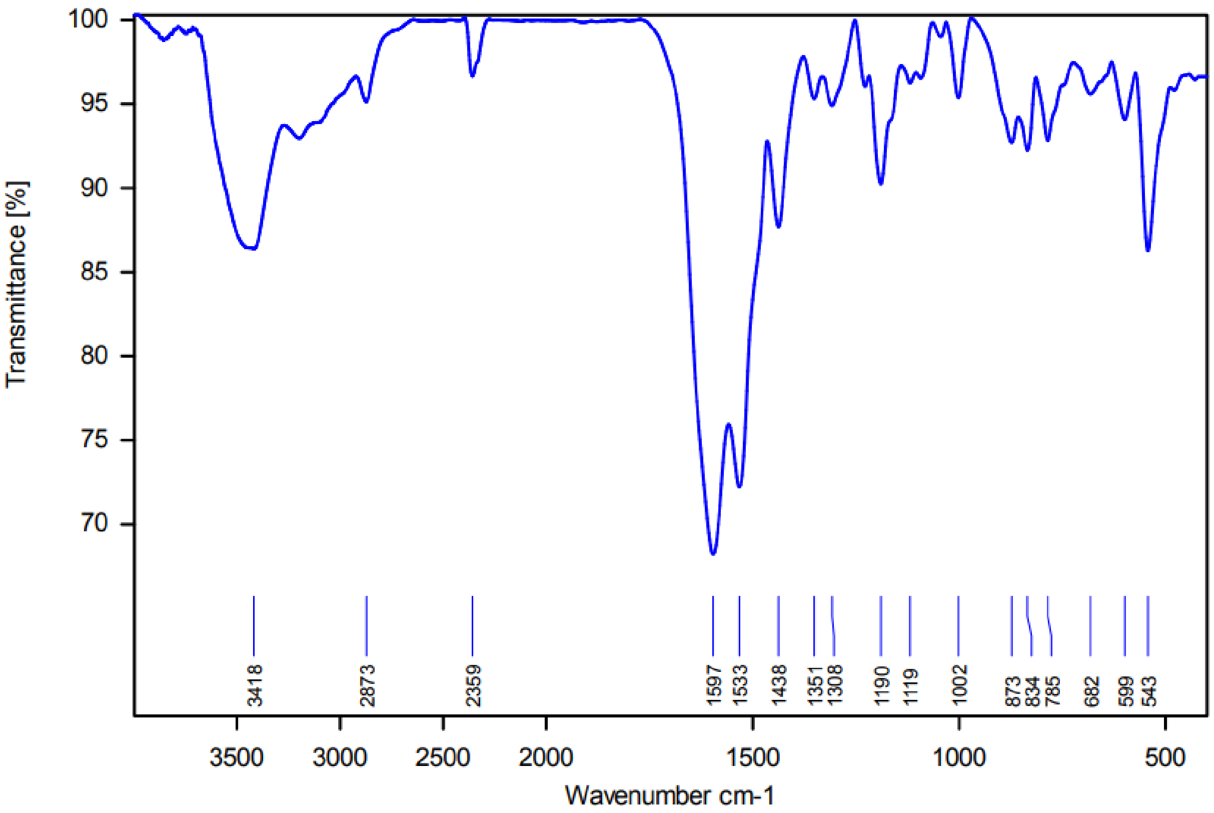

Figure S6. The FT-IR spectrum of 4-(4-chlorophenyl)-3-methyl-7-thioxo-4,6,7,8-tetrahydropyrazolo [5,6]pyrano[2,3-d]pyrimidine-5(1H)-one (2i) (Table 2, Entry 9)

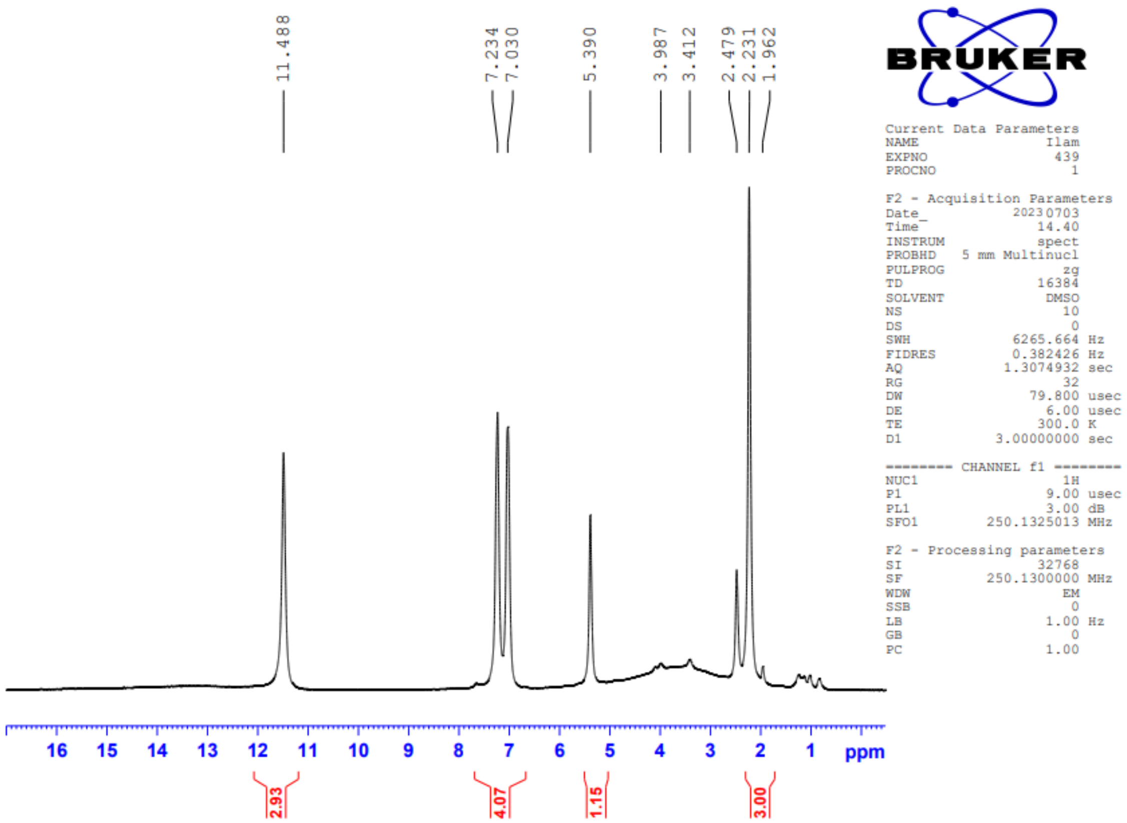

Figure S7. The ^{1}H NMR spectrum of 4-(4-chlorophenyl)-3-methyl-7-thioxo-4,6,7,8-tetrahydropyrazolo [5,6]pyrano[2,3-d]pyrimidine-5(1H)-one (2i) (Table 2, Entry 9)

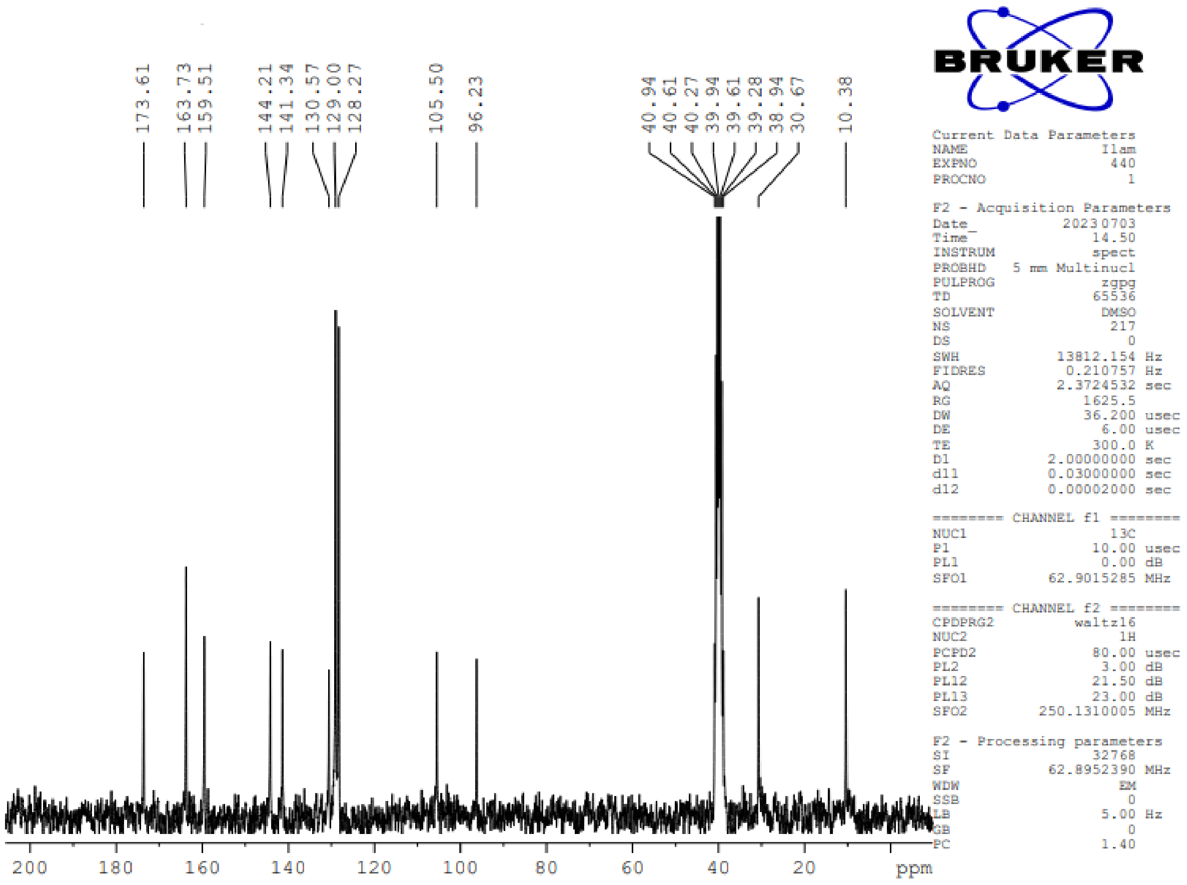

Figure S8. The ^{13}C NMR spectrum of 4-(4-chlorophenyl)-3-methyl-7-thioxo-4,6,7,8-tetrahydropyrazolo [5,6]pyrano[2,3-d]pyrimidine-5(1H)-one (2i) (Table 2, Entry 9)

3-Methyl-4-(3-nitrophenyl)-1,4-dihydropyrazolo[4’,3’:5,6]pyrano[2,3-d]pyrimidine-5,7(6H,8H)-diones (2g) (Table 2, Entry 7)

white solid; Yield: 80%; M.p. 266-268°C; IR (KBr): 3616, 3497, 3160, 2924, 1712, 1585, 1527, 1479, 1299, 1231, 1184, 548 cm^{-1}; ^{1}H NMR (250 MHz, DMSO-d6) δ (ppm): 13.24 (s, 1H), 10.27 (s, 4H), 7.99 (s, 2H), 7.85 (s, 2H), 7.51 (s, 4H), 5.52 (s, 2H), 2.25 (s, 5H); ^{13}C NMR (63 MHz, DMSO-d6) δ (ppm): 165.32, 160.47, 151.17, 148.16, 145.65, 144.29, 134.28, 129.89, 121.58, 121.20, 105.26, 91.34, 31.24, 10.46.

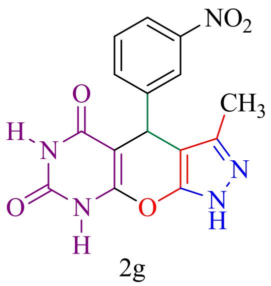

2g

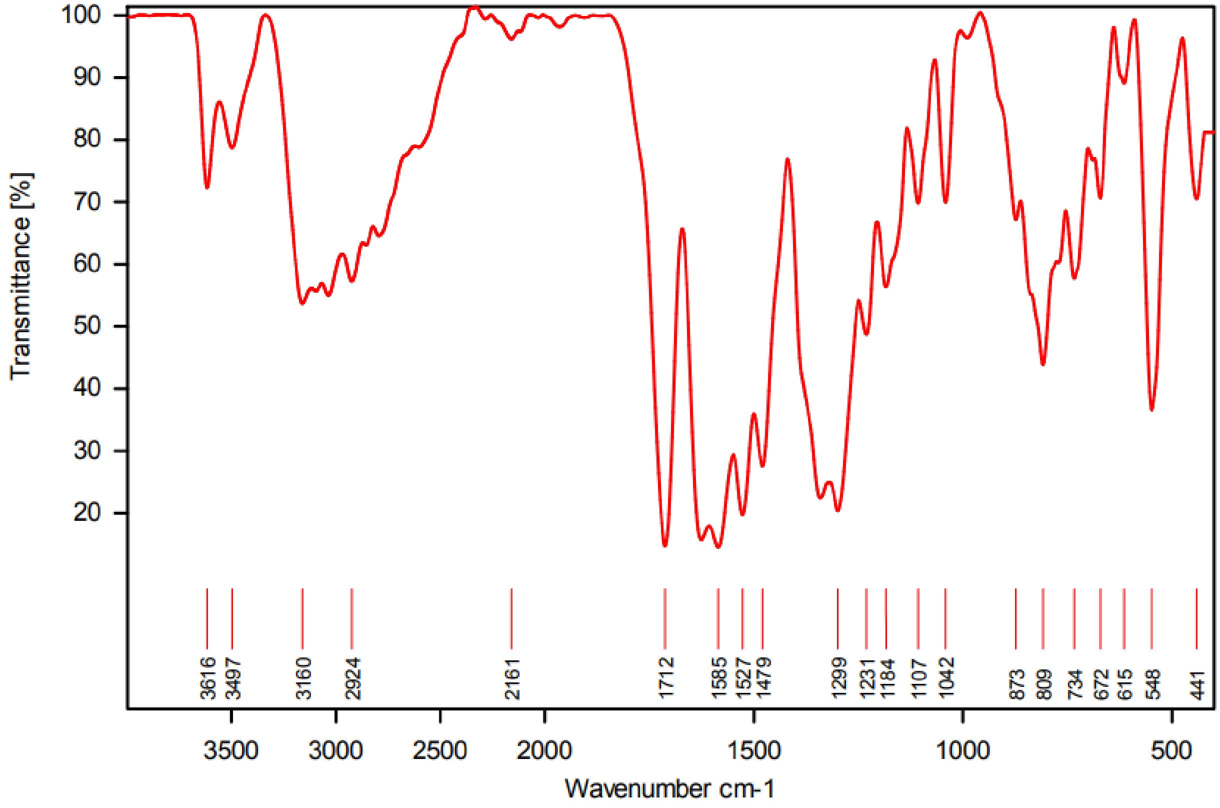

Figure S9. The FT-IR spectrum of 3-Methyl-4-(3-nitrophenyl)-1,4-dihydropyrazolo[4',3':5,6]pyrano[2,3-d]pyrimidine-5,7(6H,8H)-diones (2g) (Table 2, Entry 7)

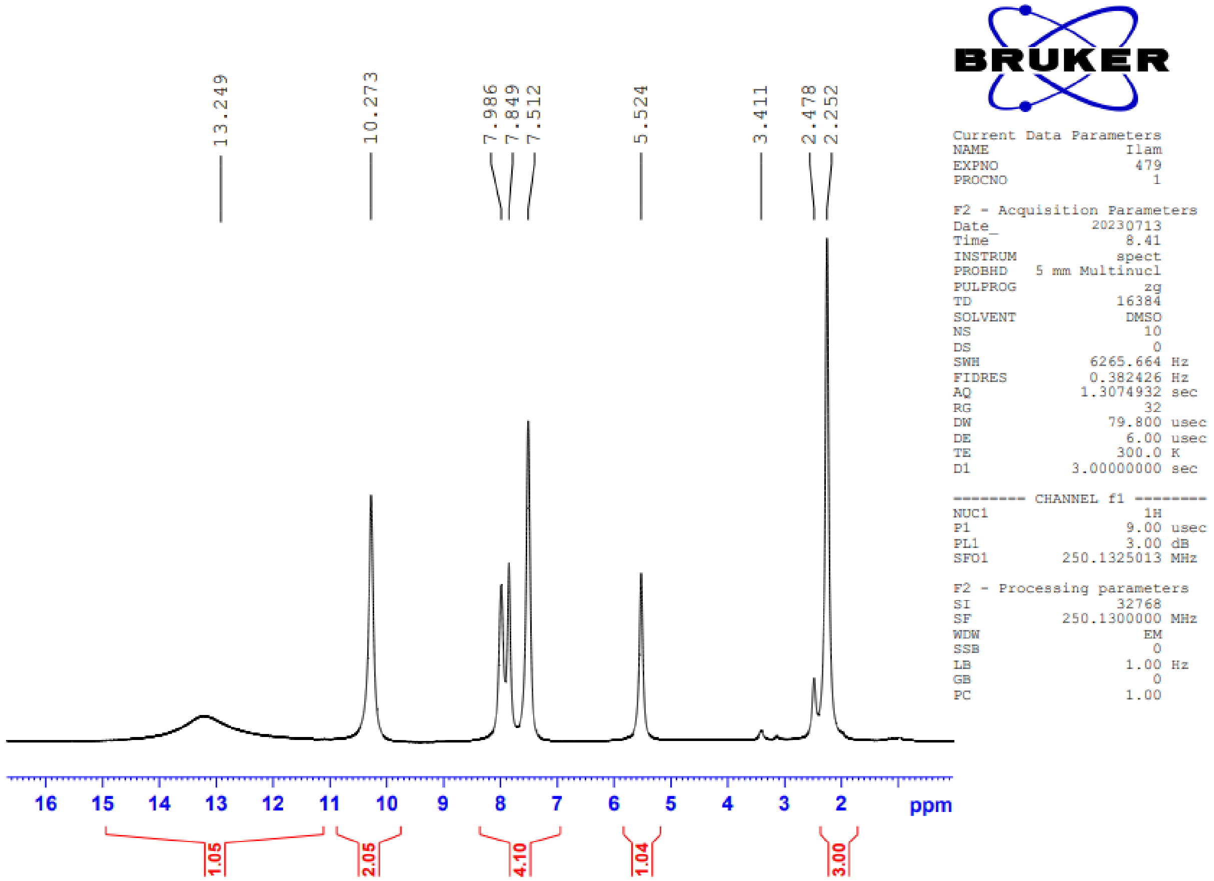

Figure S10. The ^{13}H NMR spectrum of 3-Methyl-4-(3-nitrophenyl)-1,4-dihydropyrazolo[4',3':5,6]pyrano[2,3-d]pyrimidine-5,7(6H,8H)-diones (2g) (Table 2, Entry 7)

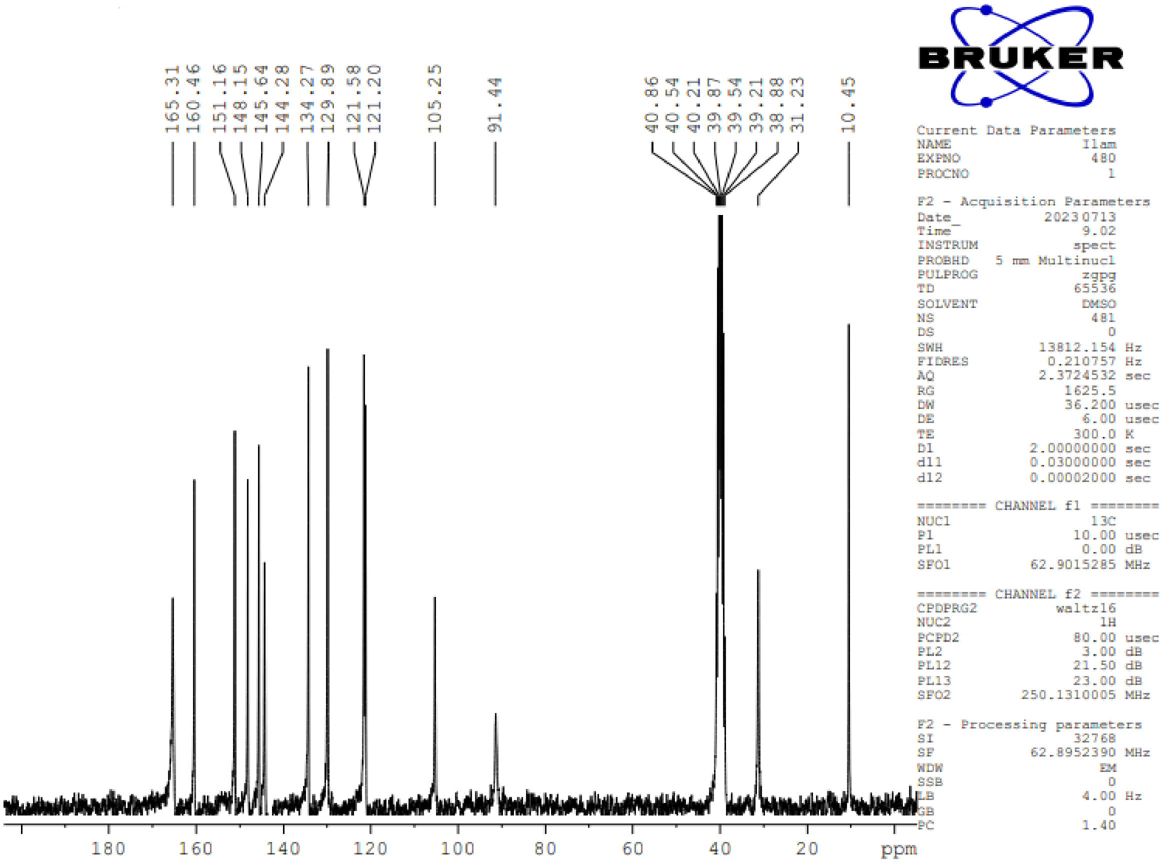

Figure S11. The ^{13}C NMR spectrum of 3-Methyl-4-(3-nitrophenyl)-1,4-dihydropyrazolo[4',3':5,6]pyrano[2,3-d]pyrimidine-5,7(6H,8H)-diones (2g) (Table 2, Entry 7)

Data on the physical properties of tetrahydrodipyrazolopyridines

3,5-Dimethyl-4-(4-chlorophenyl)-1,4,7,8-tetrahydrodipyrazolo[3,4-b;4',3'-e]pyridine (3a) (Table 4, Entry 1)

white solid; Yield: 98%; M.p. 252-254°C.

3,5-Dimethyl-4-(4-methoxyphenyl)-1,4,7,8-tetrahydrodipyrazolo[3,4-b;4',3'-e]pyridine (3c) (Table 4, Entry 3)

Cream solid; Yield: 90%; M.p. 187-190°C.

3,5-Dimethyl-4-(2-nitrophenyl)-1,4,7,8-tetrahydrodipyrazolo[3,4-b;4',3'-e]pyridine (3e) (Table 4, Entry 5)

Cream solid; Yield: 88%; M.p. 189-190°C.

3,5-Dimethyl-4-(4-bromophenyl)-1,4,7,8-tetrahydrodipyrazolo[3,4-b;4',3'-e]pyridine (3g) (Table 4, Entry 7)

Pale yellow solid; Yield: 91%; M.p. 169-170°C.

3,5-Dimethyl-4-(4-hydroxyphenyl)-1,4,7,8-tetrahydrodipyrazolo[3,4-b;4',3'-e]pyridine (3h) (Table 4, Entry 8)

white solid; Yield: 92%; M.p. 269-270°C.

3,5-Dimethyl-4-(2-chlorophenyl)-1,4,7,8-tetrahydrodipyrazolo[3,4-b;4',3'-e]pyridine (3i) (Table 4, Entry 9)

pale brown solid; Yield: 90%; M.p. 165-167°C.

3,5-Dimethyl-4-(4-nitrophenyl)-1,4,7,8-tetrahydrodipyrazolo[3,4-b;4',3'-e]pyridine (3j) (Table 4, Entry 10)

Cream solid; Yield: 80%; M.p. 298-300°C.

3,5-Dimethyl-4-(3-bromophenyl)-1,4,7,8-tetrahydrodipyrazolo[3,4-b;4',3'-e]pyridine (3k) (Table 4, Entry 11)

Cream solid; Yield: 91%; M.p. 247-248°C.

4- (1,4,7,8-Tetrahydro-3,5-dimethyldipyrazolo[3,4-b:4',3'-e] pyridin-4-yl)-N,N-dimethyl aniline (3l) (Table 4, Entry 12)

yellow solid; Yield: 90%; M.p. 240-243°C.

3,5-Dimethyl-4-(2-methoxyphenyl)-1,4,7,8-tetrahydrodipyrazolo[3,4-b;4',3'-e]pyridine (3d) (Table 4, Entry 4)

Pale yellow solid; Yield: 93%; M.p. 180-182°C; IR (KBr): 3218, 2929, 1730, 1598, 1462, 1281, 1032, 751 cm^{-1}.

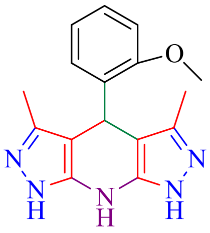

3d

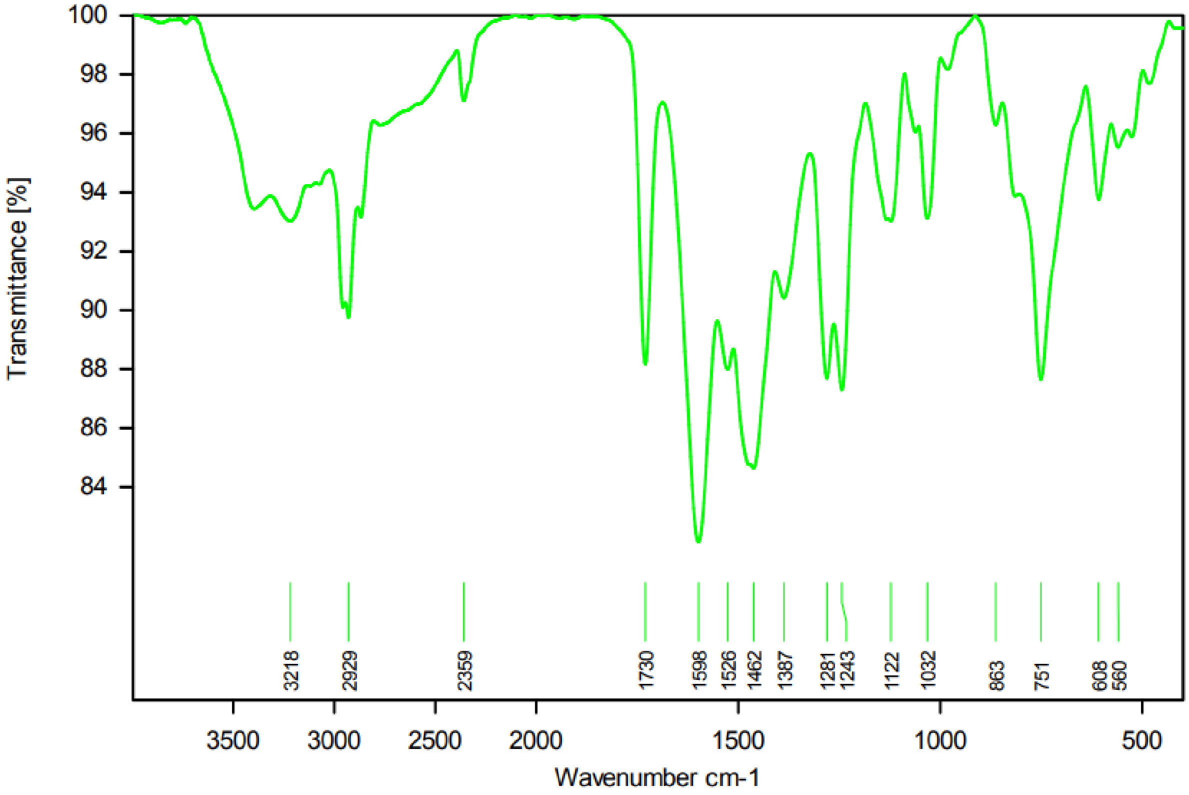

Figure S12. The FT-IR spectrum of 3,5-Dimethyl-4-(2-methoxyphenyl)-1,4,7,8-tetrahydrodipyrazolo[3,4-b;4',3'-e]pyridine (3d) (Table 4, Entry 4)

1,4,7,8-Tetrahydro-3,5-dimethyl-4-phenyldipyrazolo-[3,4- b:4',3'-e]pyridine (3b) (Table 4, Entry 2)

white solid; Yield: 98%; M.p. 241-243°C; IR (KBr): 3362, 2725, 1606, 1486, 1448, 1143, 727 cm^{-1}; ^{1}H NMR (250 MHz, DMSO-d6) δ (ppm): 11.24 (s, 4H), 7.13 (s, 5H), 4.80 (s, 1H), 2.05 (s, 7H), 1.03 (s, 1H); ^{13}C NMR (63 MHz, DMSO-d6) δ (ppm): 161.53, 143.82, 140.22, 128.14, 127.91, 125.82, 104.67, 33.21, 10.81.

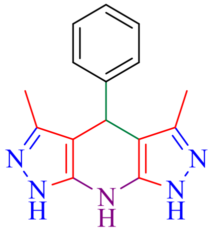

3b

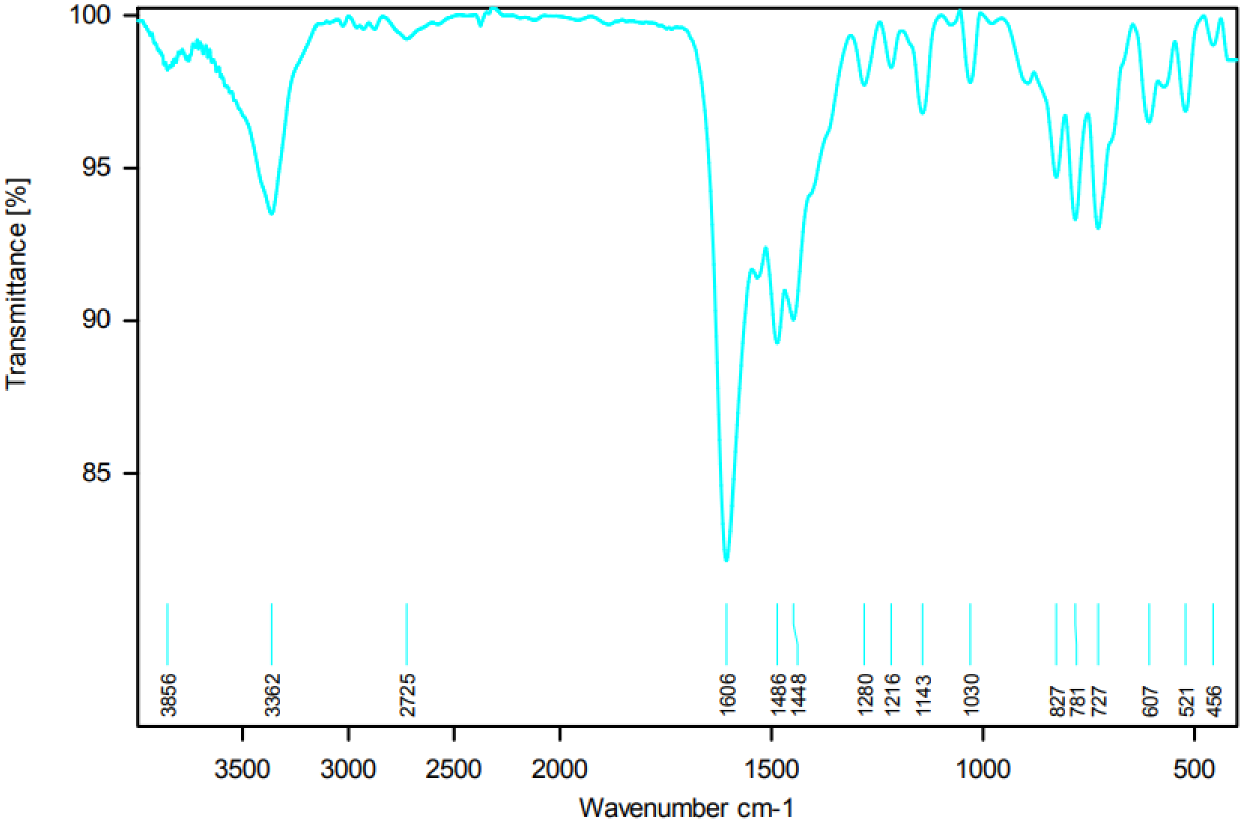

Figure S13. The FT-IR spectrum of 1,4,7,8-Tetrahydro-3,5-dimethyl-4-phenyldipyrazolo-[3,4- b:4',3'-e]pyridine (3b) (Table 4, Entry 2)

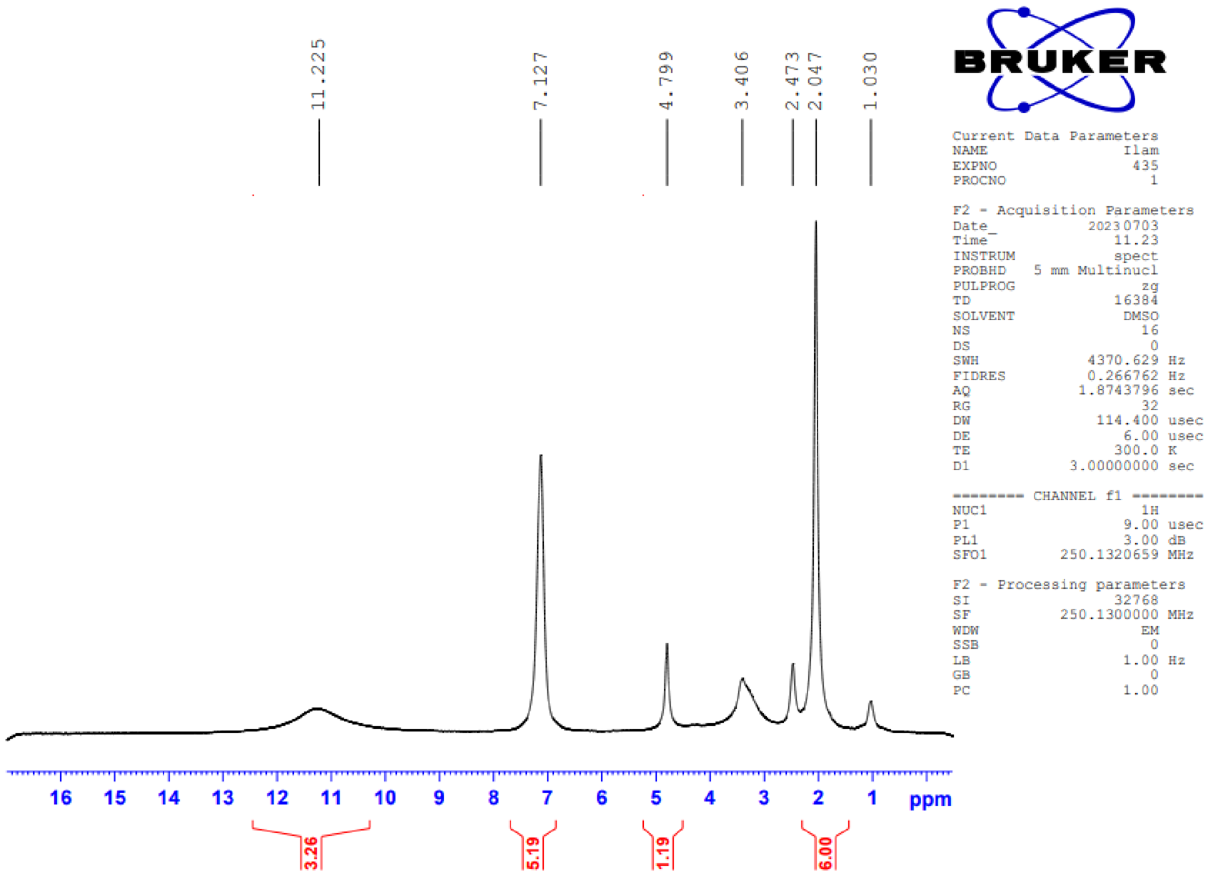

Figure S14. The ^{13}H NMR spectrum of 1,4,7,8-Tetrahydro-3,5-dimethyl-4-phenyldipyrazolo-[3,4- b:4',3'-e]pyridine (3b) (Table 4, Entry 2)

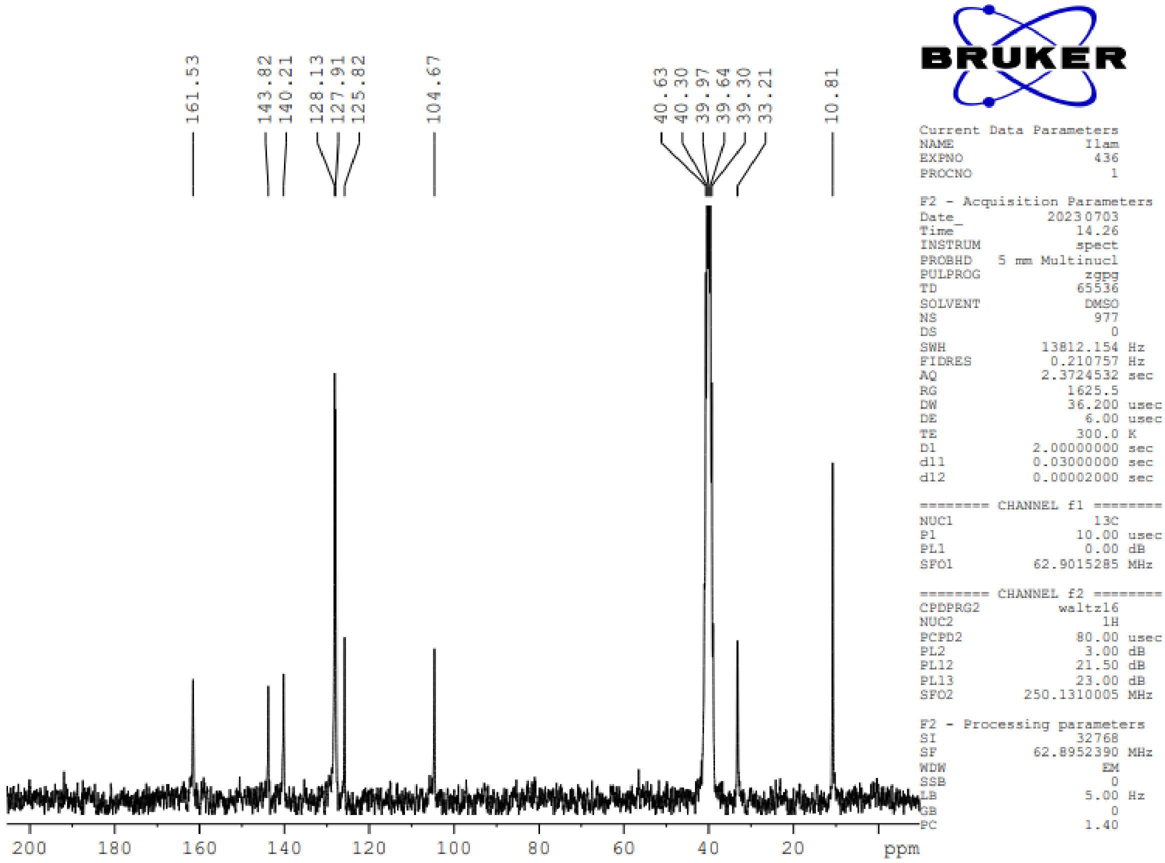

Figure S15. The ^{13}C NMR spectrum of 1,4,7,8-Tetrahydro-3,5-dimethyl-4-phenyldipyrazolo-[3,4- b:4',3'-e]pyridine (3b) (Table 4, Entry 2)

3,5-Dimethyl-4-(3-nitrophenyl)-1,4,7,8-tetrahydrodipyrazolo[3,4-b;4',3'-e]pyridine (3f) (Table 4, Entry 6)

Cream solid; Yield: 85%; M.p. 284-286°C; ^{1}H NMR (250 MHz, DMSO-d6) δ (ppm): 11.35 (s, 4H), 7.97 (d, *J* = 16.4 Hz, 2H), 7.53 (s, 2H), 4.96 (s, 1H), 2.08 (s, 5H); ^{13}C NMR (63 MHz, DMSO-d6) δ (ppm): 161.35, 146.26, 148.04, 140.34, 135.04, 129.66, 122.39, 121.14, 103.76, 33.02, 10.73.

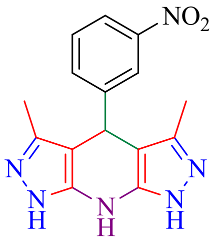

3f

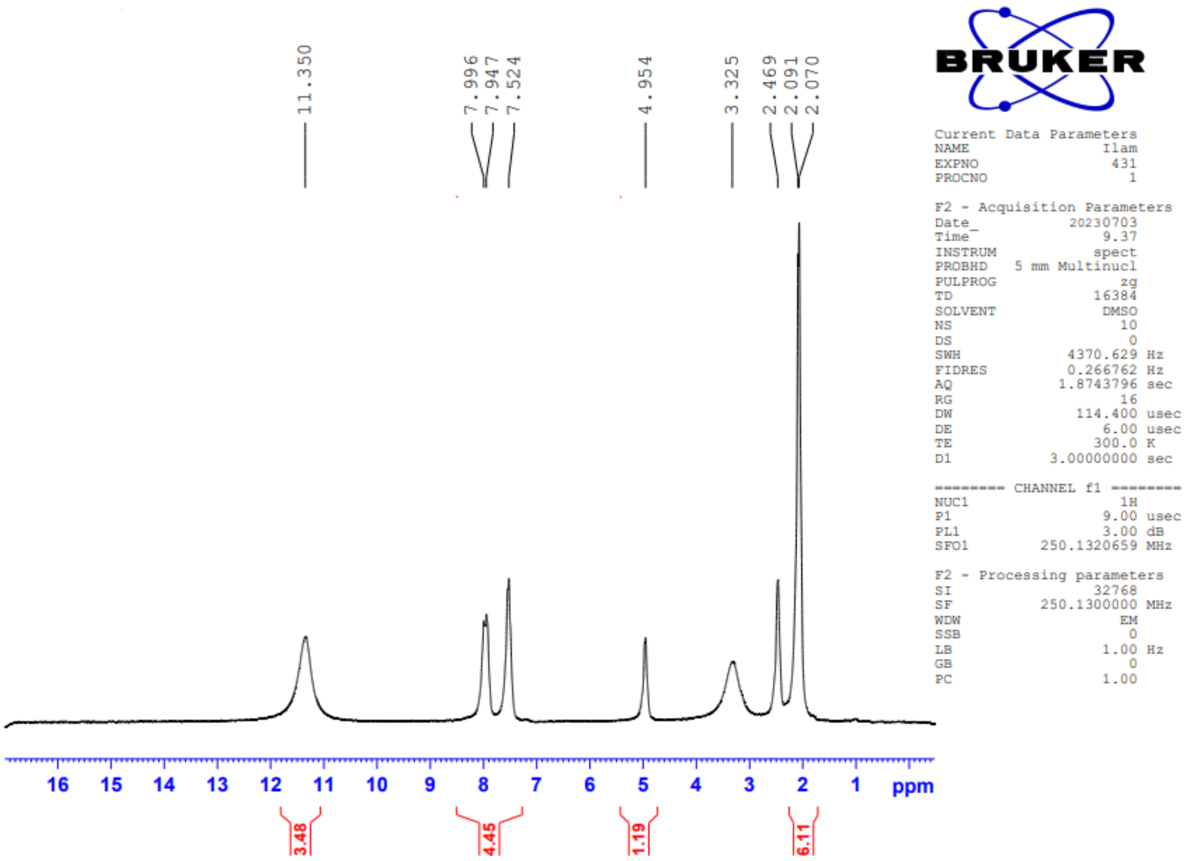

Figure S16. The ^{13}H NMR spectrum of 3,5-Dimethyl-4-(3-nitrophenyl)-1,4,7,8-tetrahydrodipyrazolo[3,4-b;4',3'-e]pyridine (3f) (Table 4, Entry 6)

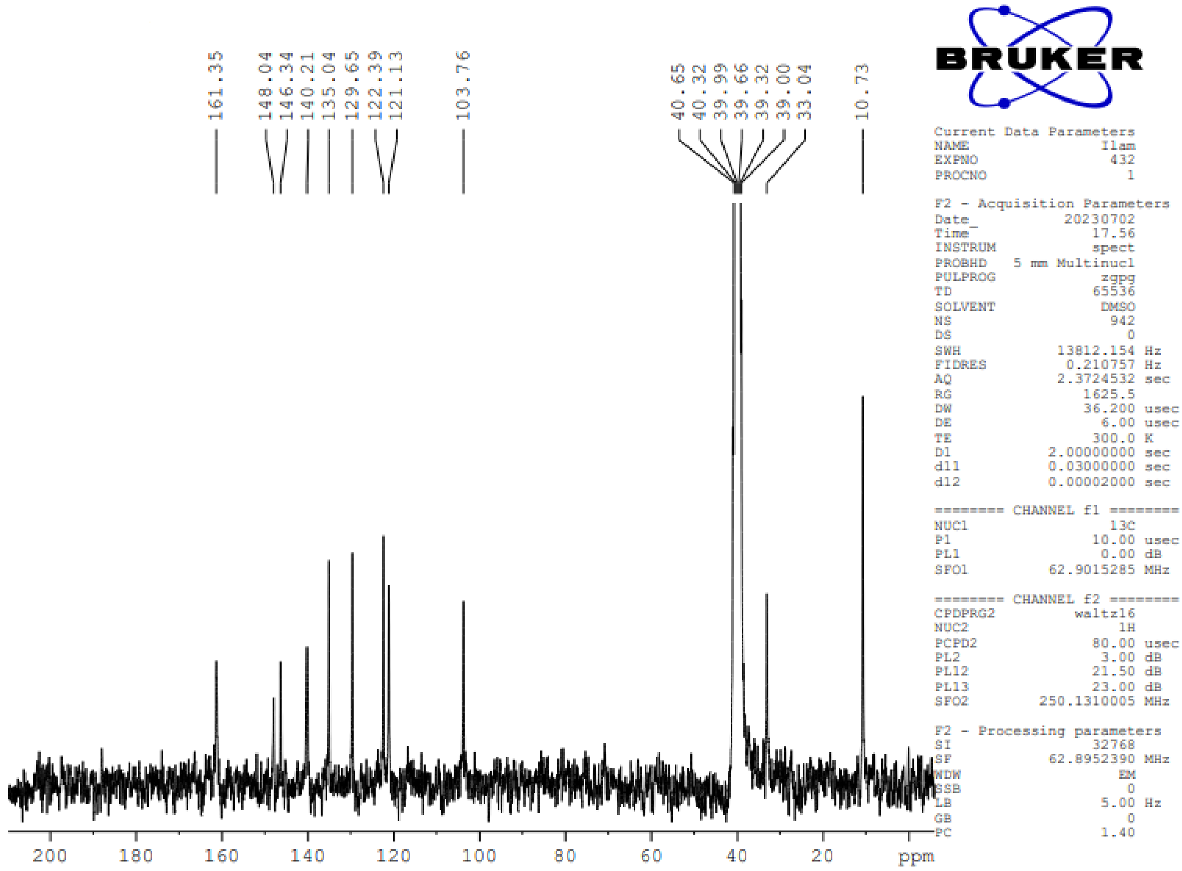

Current Data Parameters
NAME Ilam
EXPNO 432
PROCNO 1

F2 - Acquisition Parameters
Date_ 20230702
Time 17.56
INSTRUM spect
PROBHD 5 mm Multinucl
PULPROG zgpg
TD 65536
SOLVENT DMSO
NS 942
DS 0
SWH 13812.154 Hz
FIDRES 0.210757 Hz
AQ 2.3724532 sec
RG 1625.5
DW 36.200 usec
DE 6.00 usec
TE 300.0 K
D1 2.00000000 sec
d11 0.03000000 sec
d12 0.00002000 sec

======== CHANNEL f1 ========
NUC1 13C
P1 10.00 usec
PL1 0.00 dB
SFO1 62.9015285 MHz

======== CHANNEL f2 ========
CPDPRG2 waltz16
NUC2 1H
PCPD2 80.00 usec
PL2 3.00 dB
PL12 21.50 dB
PL13 23.00 dB
SFO2 250.1310005 MHz

F2 - Processing parameters
SI 32768
SF 62.8952390 MHz
WDW EM
SSB 0
LB 5.00 Hz
GB 0
PC 1.40

Figure S17. The ^{13}C NMR spectrum of 3,5-Dimethyl-4-(3-nitrophenyl)-1,4,7,8-tetrahydrodipyrazolo[3,4-b;4',3'-e]pyridine (3f) (Table 4, Entry 6)

1,4-Bis[(1,4,7,8-Tetrahydro-3,5-dimethyldipyrazolo[3,4-b:40 ,30 -e]pyridin-4-yl)] benzene (3m) (Table 4, Entry 13)

Pale orange solid; Yield: 96%; M.p. >300 °C; IR (KBr): 3401, 1602, 1457, 1215, 1136, 785, 593 cm^{-1}; ^{1}H NMR (250 MHz, DMSO-d6) δ (ppm): 11.31 (s, 3H), 6.93 (s, 2H), 4.68 (s, 1H), 2.03 (s, 7H), 1.02 (s, 2H); ^{13}C NMR (63 MHz, DMSO-d6) δ (ppm):168.25, 161.65, 140.36, 127.23, 109.50, 32.81, 10.79.

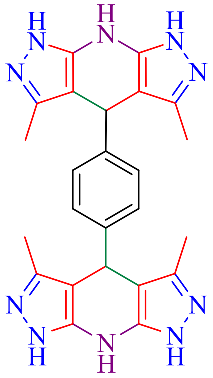

3m

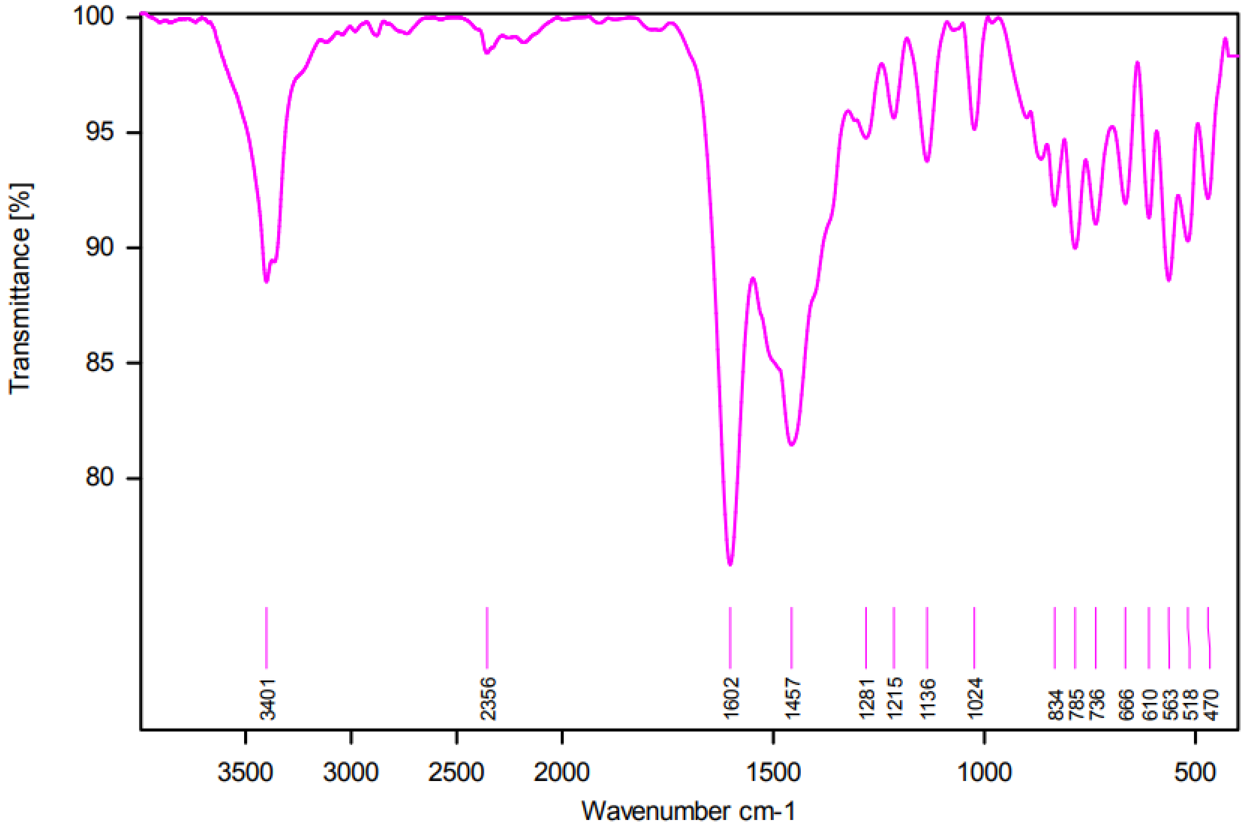

Figure S18. The FT-IR spectrum of 1,4-Bis[(1,4,7,8-Tetrahydro-3,5-dimethyldipyrazolo[3,4-b:40 ,30 -e]pyridin-4-yl)] benzene (3m) (Table 4, Entry 13)

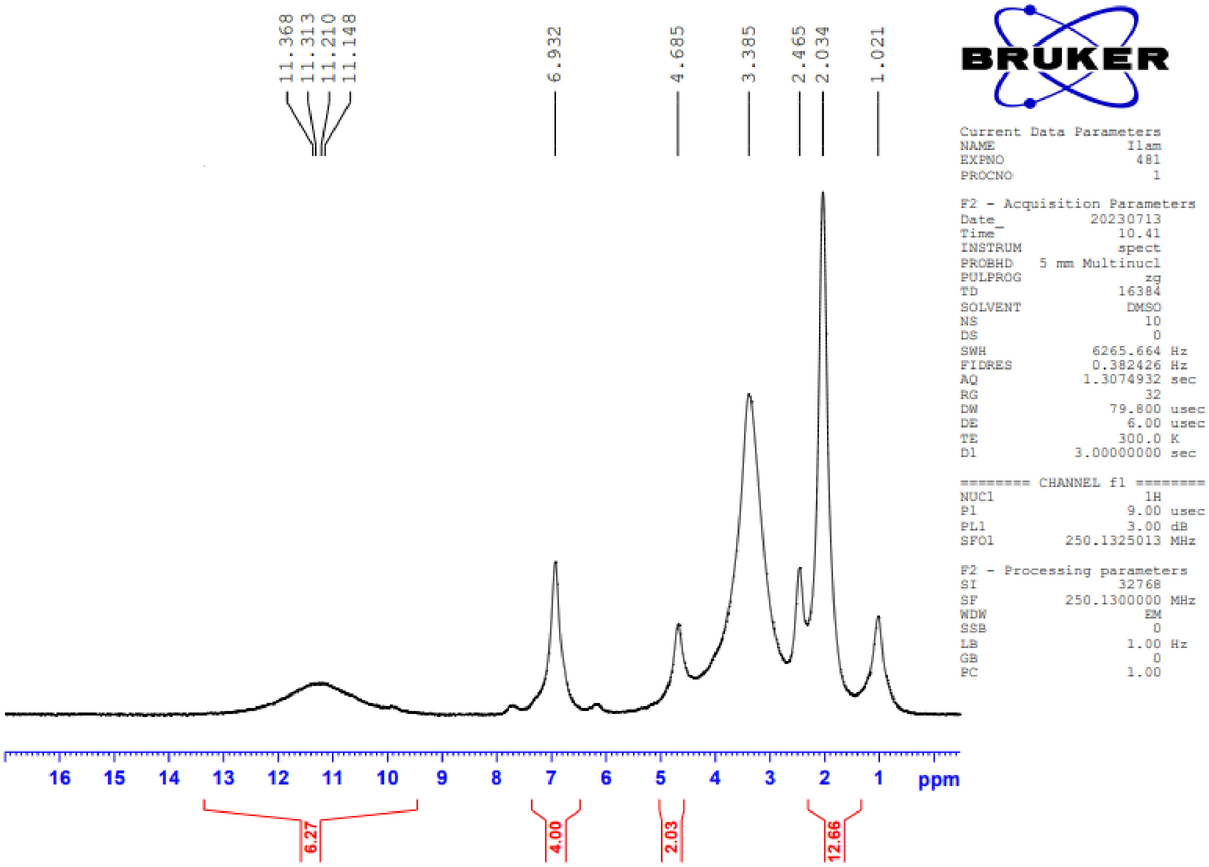

Figure S19. The ^{13}H NMR spectrum of 1,4-Bis[(1,4,7,8-Tetrahydro-3,5-dimethyldipyrazolo[3,4-b:40 ,30 -e]pyridin-4-yl)] benzene (3m) (Table 4, Entry 13)

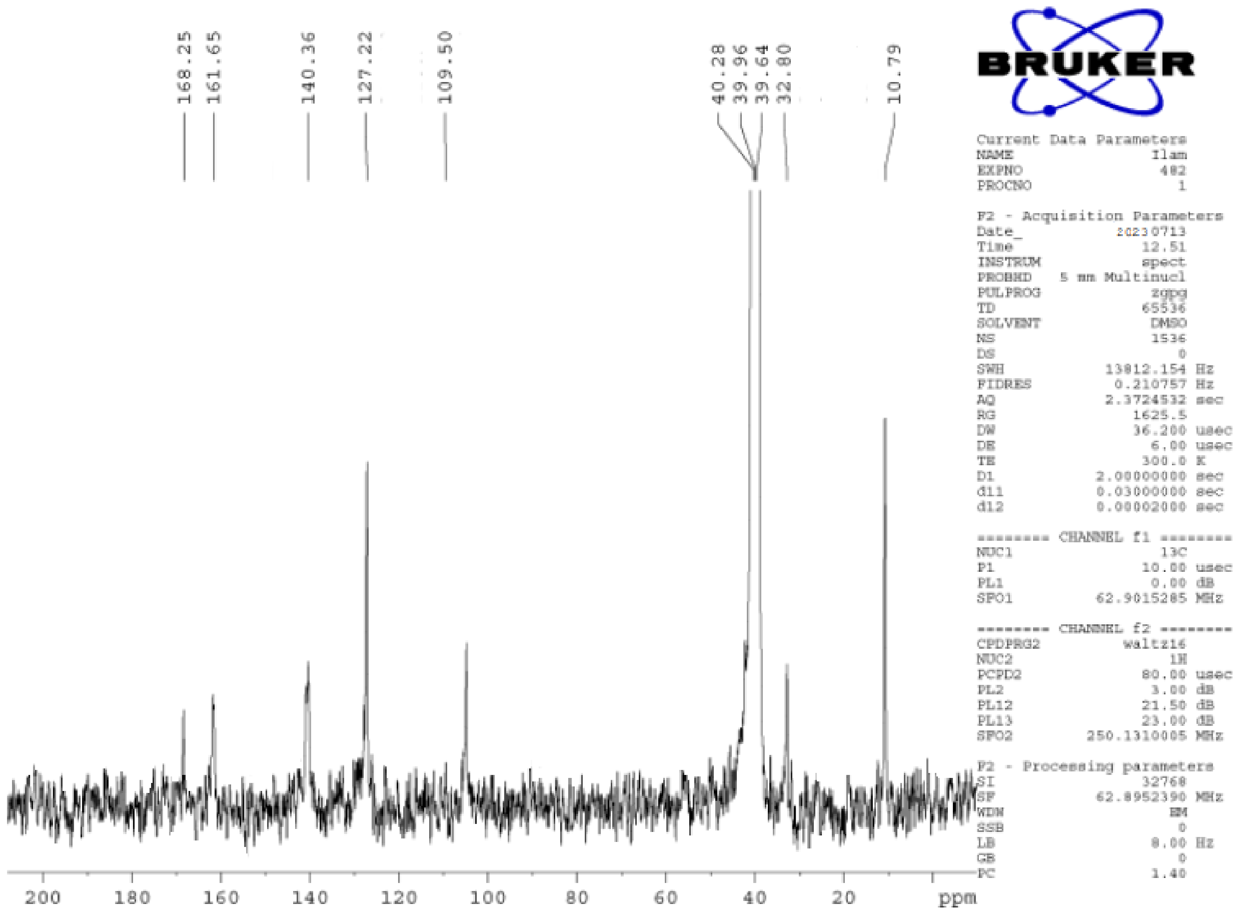

Figure S20. The ^{13}C NMR spectrum of 1,4-Bis[(1,4,7,8-Tetrahydro-3,5-dimethyldipyrazolo[3,4-b:40 ,30 -e]pyridin-4-yl)] benzene (3m) (Table 4, Entry 13)
